# SpoVG is an important regulator of sporulation and affects biofilm formation by regulating Spo0A transcription in *Bacillus cereus* 0–9

**DOI:** 10.1186/s12866-021-02239-6

**Published:** 2021-06-08

**Authors:** Qiubin Huang, Zhen Zhang, Qing Liu, Fengying Liu, Yupeng Liu, Juanmei Zhang, Gang Wang

**Affiliations:** 1 Institute of Microbial Engineering, Laboratory of Bioresource and Applied Microbiology, School of Life Sciences, Henan University, Jinming Street, Kaifeng, 475004 China; 2 Engineering Research Center for Applied Microbiology of Henan Province, Kaifeng, 475004 China; 3 School of Pharmaceutical, Henan Univeristy, Kaifeng, 475004 China

**Keywords:** Biofilm, SpoVG, Sporulation, SinI/R, Spo0A, *Bacillus cereus*

## Abstract

**Background:**

*Bacillus cereus* 0–9, a Gram-positive, endospore-forming bacterium isolated from healthy wheat roots in our previous research, is considered to be an effective biocontrol strain against several soil-borne plant diseases. SpoVG, a regulator that is broadly conserved among many Gram-positive bacteria, may help this organism coordinate environmental growth and virulence to survive. This study aimed to explore the multiple functions of SpoVG in *B. cereus* 0–9.

**Methods:**

The gene knockout strains were constructed by homologous recombination, and the sporulation process of *B. cereus* 0–9 and its mutants were observed by fluorescence staining method. We further determined the spore yields and biofilm formation abilities of test strains. Transcriptional fusion strains were constructed by overlapping PCR technique, and the promoter activity of the target gene was detected by measuring its fluorescence intensity**.** The biofilm production and colonial morphology of *B. cereus* 0–9 and its mutants were determined to study the functions of the target genes, and the transcription level of the target gene was determined by qRT-PCR.

**Results:**

According to observation of the sporulation process of *B. cereus* 0–9 in germination medium, SpoVG is crucial for regulating sporulation stage V of *B. cereus* 0–9, which is identical to that of *Bacillus subtilis* but differs from that of *Bacillus anthracis*. In addition, SpoVG could influence biofilm formation of *B. cereus* 0–9. The transcription levels of two genes closely related to biofilm-formation, *sipW* and *calY*, were downregulated in a Δ*spoVG* mutant. The role of SpoVG in regulating biofilm formation was further explored by deleting the genes *abrB* and *sinR* in the Δ*spoVG* mutant, respectively, generating the double mutant strains Δ*spoVG*Δ*abrB* and Δ*spoVG*Δ*sinR*. The phenotypes of these double mutants were congruent with those of the single *abrB* and *sinR* deletion strains, respectively, which showed increased biofilm formation. This indicated that *spoVG* was located upstream of *abrB* and *sinR* in the regulatory pathway of *B. cereus* biofilm formation. Further, the results of qRT-PCR and the luminescence intensity of transcriptional fusion strains indicated that *spoVG* gene deletion could inhibit the transcription of Spo0A.

**Conclusions:**

SpoVG, an important regulator in the sporulation of *B. cereus*, is located upstream of Spo0A and participates in regulation of biofilm formation of *B. cereus* 0–9 through regulating the transcription level of *spo0A*. Sporulation and biofilm formation are crucial mechanisms by which bacteria respond to adverse conditions. SpoVG is therefore an important regulator of Spo0A and is crucial for both sporulation and biofilm formation of *B. cereus* 0–9. This study provides a new insight into the regulatory mechanism of environmental adaptation in bacteria and a foundation for future studies on biofilm formation of *B. cereus*.

**Supplementary Information:**

The online version contains supplementary material available at 10.1186/s12866-021-02239-6.

## Introduction

Bacteria have developed various mechanisms, some not yet known, to adapt and respond to adverse environmental factors during the long course of evolution. Biofilm formation and sporulation are two different effective survival strategies that enable bacteria to utilize a greater variety of nutrients, endure rapid environmental changes, and resist multiple adverse threats [1]. For *Bacillus cereus*, sporulation or biofilm formation is an effective self-protective behavior used to survive environmental pressures, such as nutrient deficiency, high temperature, high salt, antibiotics, etc. *B. cereus* produces a variety of biofilms that differ in their architecture and mechanism of formation, possibly reflecting an adaptation to various adverse environments [2]. *B. cereus* can also form dormant spores under extremely adverse conditions, germinating when external environmental conditions are permissible for growth.

In *Bacillus subtilis*, sporulation is a developmental pathway in which sequential, compartmentalized gene expression is achieved by interlocking cascades of regulatory factors and morphological cues [3]. Sporulation of *B. subtilis* has been divided into so-called “stages” 0 to VII using electron microscopy. In the *B. cereus* group (which includes *Bacillus anthracis*), sporulation is comparable to that in *B. subtilis*, but there are some differences between different strains [4]. For example, spore-coat assembly begins at the mother-cell-proximal pole of the forespore in *B. subtilis*, whereas coat material first appears on the long axis of the forespore in *B. anthracis* and *B. cereus* [5]. The primary environmental signal for initiation of sporulation is nutrient limitation [6], which could also induce characteristics of other adaptive responses, such as slow growth, non-proliferative stationary-phase cells, or biofilm formation.

Biofilm formation requires a complex regulatory pathway that coordinates gene expression with external environmental conditions [3, 7, 8]. In *B. subtilis*, a matrix component operon of *tapA*-*sipW*-*tasA* is required for biofilm formation. TapA is encoded by *tapA*, in this operon, with *sipW* encoding a signal peptidase required for the secretion of both TasA and TapA [9]. The control of biofilm formation is dependent on a molecular switch comprising two transcription repressors and two anti-repressors [10]. Central to this regulatory switch is the repressor SinR [11], a DNA binding protein that represses the transcription of the *tapA*-*sipW*-*tasA* operon involved in both production of an exopolysaccharide [12] and the protein component of the matrix. SinR activity is controlled by the relative levels of various antagonists, such as SinI [13], which binds to SinR and causes de-repression because the SinI-SinR complex is no longer able to bind to DNA [14, 15]. In *B. subtilis*, biofilm formation can be triggered by the master transcription factor Spo0A, which activates biofilm formation by increasing production of SinI [16]. AbrB was also reported to play a role in the regulation of biofilm formation in *B. subtilis*. AbrB negatively regulates biofilm formation of *B. subtilis*, which is regulated negatively by Spo0A [17]. In contrast, the regulatory mechanisms that control biofilm formation of *B. cereus* are poorly understood because very few genes involved in biofilm formation have been characterized in this species.

SpoVG was initially identified in *B. subtilis* and is involved in sporulation via an unknown mechanism [18, 19], although a recent report indicates that SpoVG is a pleiotropic regulatory factor in sporulation [4]. The *spoVG* gene is highly conserved in bacteria. In *B. subtilis*, SpoVG negatively regulates asymmetric septum formation and positively regulates cortex formation. In *Staphylococcus aureus*, which is a nonsporulating bacterium, SpoVG is the main effector molecule of the *yabJ*-*spoVG* operon [3]. It is regulated by σ^B^ and can affect the formation of capsular polysaccharides, regulate the expression of virulence factors, and generate antibiotic resistance [20–22]. In *Borrelia burgdorferi*, SpoVG is a highly conserved, DNA-binding protein that interacts with *cap*5, *fmtB*, *exsA*, and *lukED* promoters [23]. According to whole genome sequencing results (GenBank: CP042874.1) and subsequent gene function annotation, the *B. cereus* 0–9 genome contains a gene whose product is an ortholog of SpoVG (locus_tag: FRY47_00280). The amino acid sequence of this SpoVG ortholog was subjected to a blast search on the National Center of Biotechnology Information (NCBI) website and was found to share 90.72% homology with that of *B. subtilis* (SpoVG_168_), and 100% sequence identity to SpoVG of *B. anthracis* (SpoVG_a_). Consequently, the orthologous protein of SpoVG is considered a product of *spoVG* in *B. cereus* 0–9. However, it has not been reported whether biofilm formation of *B. cereus* can be regulated by SpoVG, and how SpoVG participates in responding to environmental stresses. This study explored the role of SpoVG in the environmental adaptation of *B. cereus* by investigating SpoVG involvement in regulation of sporulation and biofilm formation of the biocontrol strain *B. cereus* 0–9.

## Materials and methods

### Strains, plasmids, culture media, and growth conditions

The properties and culture conditions of *B. cereus* 0–9 were reported in our previous study [24]. *Escherichia coli* 116 (*pir*+) used for the propagation of plasmids was incubated in Lysogeny Broth (LB) at 37 °C overnight. The recombinant plasmid pMADchi was used in the allelic exchange method for generating complementary mutants [25]. All strains and plasmids used in this study are listed in Table 1. Spore yield was measured in modified germination medium (MG) comprising 1 g glucose, 7 g K_2_HPO_4_, 3 g KH_2_PO_4_, 0.2 g yeast extract, 0.1 g MgSO_4_·7H_2_O, 0.01 g CaCl_2_, 0.001 g FeSO_4_, 0.1 g NaCl and distilled water to 1 L. For biofilm formation, tested strains were cultured in modified Minimal salts glycerol glautamate (MSgg) medium comprising 100 mM 3-(N-morpholine) propyl sulfonic acid, 2 mM MgCl_2_, 700 μM CaCl_2_, 50 μM FeCl_2_, 50 μM MnCl_2_, 5 mM phosphate buffer, 2 μM vitamin B_1_, 1 μM ZnCl_2_, 5 g/L yeast powder, and 5 g/L glycerol. MSgg solid media, made by adding 2% agar powder, was used to observe colony morphology of the tested strains. Nutrient agar (NA) plate was made of 10 g peptone, 5 g yeast extracts, 5 g NaCl, 20 g agar and distilled water to 1 L.

**Table 1:** Tested strains and plasmids used in this experiment

Name	Properties and Application	Source
Strains		
*B. cereus* 0–9	Wild type strain in this study	Kept in our laboratory, isolated from wheat root.
*E. coli* 116 (*pir*+)	Plasmid propagation	Purchased from BioVector NTCC
*E. coli* GM2163 (dam-)	Demethylation	Purchased from BioVector NTCC
HQ1021	The *spoVG* gene defect mutant, ∆*spoVG*	Construct in this study
ZY1001	The *abrB* gene defect mutant, ∆*abrB*	Construct in this study
FPU1061I	The *sinI* gene defect mutant, ∆*sinI*	Construct in this study
FPU1062R	The *sinR* gene defect mutant, ∆*sinR*	Construct in this study
ZL1002	The *spo0A* gene defect mutant, ∆*spo0A*	Construct in this study
HQ2021	The double knockout strain of *spoVG* and *sinI* genes, ∆*spoVG*∆*sinI*	Construct in this study
HQ2022	The double knockout strain of *spoVG* and *sinR* genes, ∆*spoVG*∆*sinR*	Construct in this study
HQ2023	The double knockout strain of *spoVG* and *abrB* genes, ∆*spoVG*∆*abrB*	Construct in this study
HQ2024	The double knockout strain of *spoVG* and *spo0A* genes, ∆*spoVG*∆*spo0A*	Construct in this study
HQ7110	Δ*spoVG* supplemented with its native *spoVG* gene by allelic exchange, ∆*spoVG*::*spoVG*	Construct in this study
HQ7170	Δ*spoVG* supplemented with *spoVG* gene from *B. subtilis* 168 by allelic exchange, ∆*spoVG*::*spoVG*_168_	Construct in this study
HQ7121	HQ1021 supplemented with its native *spo0A* gene by reverse complementary, ∆*spoVG*/*spo0A*	Construct in this study
HQ7221	HQ2024 supplemented with its native *spo0A* gene by reverse complementary, ∆*spoVG*∆*spo0A*/*spo0A*	Construct in this study
HQ7222	HQ2024 supplemented with its native *spoVG* gene by reverse complementary, ∆*spoVG*∆*spo0A*/*spoVG*	Construct in this study
HQg6071	*B. cereus* 0–9 with P*exsY*-GUS labeling	Construct in this study
HQg6171	HQ1021 (Δ*spoVG*) with P*exsY*-GUS labeling	Construct in this study
HQg6072	Wild *B. cereus* 0–9 with P*sipW*-GFP labeling	Construct in this study
HQg6172	HQ1021 with P*sipW*-GFP labeling	Construct in this study
HQg6073	Wild *B. cereus* 0–9 with P*calY*-GFP labeling	Construct in this study
HQg6173	HQ1021with P*calY*-GFP labeling	Construct in this study
HQg6074	Wild *B. cereus* 0–9 with P*abrB*-GFP labeling	Construct in this study
HQg6174	HQ1021 with P*abrB*-GFP labeling	Construct in this study
HQg6175	Wild *B. cereus* 0–9 with P*sinI*-GFP labeling	Construct in this study
HQg6175	HQ1021 with P*sinI*-GFP labeling	Construct in this study
Plasmid		
pAD	For gene knockout	Takara, Dalian
*spoVG*-pMAD	For gene knockout, Amp^+^; Erm^+^	Construct in this study
*abrB*-pMAD	For gene knockout, Amp^+^; Erm^+^	Construct in this study
pAD-pgal-JT	For reverse complementation	Stored in our laboratory
pMAD-*chi*	For gene complementation, Cm^+^	Construct in this study
pET28a*-gapB*	Expression of protein, Km^+^	Construct in this study

### Sequence data analysis and genetic distance calculation

Whole-genome sequencing (GenBank: CP042874.1) and subsequent gene function annotation revealed that the *B. cereus* 0–9 genome encodes a SpoVG protein (Protein ID: QEF19539.1). The putative amino acid sequences used in this study were downloaded from the GenBank nucleotide sequence database. Sequence alignment of SpoVG proteins was performed in the DNAMAN program (version 5.2.2.0). BLAST (version 2.10.1) was used to search the GenBank database for SpoVG homologues in other species of bacteria and their amino acid sequences in fasta format were downloaded and inputted into DNAMAN software for multiple sequence alignment.

### Construction of gene deletion strains and complemented strains

The *spoVG*-knockout mutant strain ∆*spoVG* of *B. cereus* 0–9 (HQ1021) was constructed by a previously described allelic exchange method [26]. In detail, two fragments suitable for allelic exchange were created by cloning two *BamH*I-*Xho*I DNA fragments containing locus-specific flanking regions into the *BamH*I site of the pMAD vector. These fragments were generated via PCR using the primers in Table S1. Ligation of the two fragments led to precise deletion of the respective open reading frame from the start to the stop codons and to generation of an *Xho*I site at the locus site. The same procedure was used to create ∆*sinI*, ∆*sinR*, ∆*abrB*, ∆*spo0A*, ∆*spoVG*∆*sinI*, ∆*spoVG*∆*abrB*, and ∆*spoVG*∆*spo0A* mutants of *B. cereus* 0–9. A pMADchi vector was constructed using the plasmid pMAD with a *chi* gene from *B. cereus* 0–9, and was used to construct complementation strains [25]. Briefly, after PCR amplification and retrieval, the target gene fragments were digested and inserted into the recombinant pMADchi vector, which was then used to generate the complemented mutant strain by allelic exchange. All strains constructed in this study are shown in Table 1.

### Determination of spore yield

The spore ratios of *B. cereus* 0–9 and its mutants were determined using the previously reported method of Zhang et al. [24] In brief, each strain cultured in 50 mL MG medium at 30 °C and 220 rpm to exponential phase (OD_600_ up to 0.8), and samples collected every 2 hours. One milliliter of each sample suspension was taken, where 0.5 mL was used to determine the count of spore yield and the other 0.5 mL was immediately heated at 80 °C for 10 min. Following incubation, the cultures were spread on LB plates without dilution, and incubated overnight at 30 °C. The following morning, colonies were counted. The sporulation state of each sample was observed under a microscope after staining spores using the classical Schaeffer-Fulton staining method [25]. The sporulation stage in which SpoVG played a key role was determined using two fluorescent dyes to stain the cell membranes and spores, respectively. 4′,6-Diamidino-2-phenylindole (DAPI) binds specifically to DNA and stains the nucleus with blue fluorescence; FM4–64 binds to neutral cell membranes and appears as red fluorescence under a fluorescence microscope.

### Construction of transcriptional fusion strains

The transcriptional fusion vector was constructed using the plasmid pMADchi. Appropriate primers were designed based on known gene sequences. The promoter fragment of the *exsY* gene and the open reading frame of the *gus* gene were amplified by PCR, respectively, and used as templates for overlapping PCR amplification to obtain the complete fragment P*spoIIR*-*gus* and P*exsY*-*gus*. After enzyme digestion by *EcolR*I and *Xho*I, the P*spoIIR*-*gus* or P*exsY*-*gus* fragment was inserted into pMADchi at *EcolR*I and *Xho*I sites to generate the recombinant plasmid P*spoIIR*-*gus*-pMAD*chi* and P*exsY*-*gus*-pMADchi, respectively. The promoter fragment of the *abrB* gene and a green fluorescent protein (GFP) fragment were amplified by PCR, respectively, and used as templates for overlapping PCR amplification to obtain the complete fragment P*abrB*-GFP. The P*abrB*-GFP fragment was inserted into pMADchi at *EcolR*I and *Xho*I restriction enzyme sites to generate the recombinant plasmid P*abrB*-*gfp*-pMADchi. The same method was employed to construct transcriptional fusion vectors P*sipW*-*gfp*-pMADchi, P*calY*-*gfp*-pMADchi, P*sinI*-*gfp*-pMADchi and P*spo0A*-*gfp*-pMADchi. Using the previously described allelic exchange method [9], these transcriptional fusion vectors were transferred into *B. cereus* 0–9 and Δ*spoVG* mutants, respectively, to construct the mutant strains with fluorescent label. All mutant strains constructed in this study are shown in Table 1.

GUS protein is a glucuronidase that can react with the substrate 4-methylumbelliferyl beta-D-glucuronide dihydrate (MUG) to produce fluorescent 4-methylumbelliferyl (MU). MU content was measured by fluorescence spectrophotometry (Promega GloMax, USA) with an excitation wavelength of 365 nm and emission wavelength of 465 nm. Measuring fluorescence intensity reflects the expression of the *exsY* promoter. Enzyme activity (1 U) of GUS was defined as the change of fluorescence intensity per unit of protein per hour. The fluorescence intensity of GFP protein was measured by fluorescence spectrophotometry (Promega GloMax, USA) with an excitation wavelength of 488 nm and emission wavelength of 510 nm.

### Growth curve measurements

Growth curves of *B. cereus* strains in LB and MSgg medium were determined by inoculating a single bacterial colony into 5 mL LB medium and culturing overnight at 37 °C with shaking. One milliliter of overnight culture medium was centrifuged and the collected bacteria were washed three times with sterile water then re-suspended with different volumes of sterile water to ensure the OD_600_ of the tested strains was consistent. The suspensions were inoculated into the test medium at a ratio of 1:100 and were cultured in a Bioscreen C (Oy Growth Curves Ab Ltd., Finland) at 37 °C with 30 min intervals to automatically record the growth curves of the tested strains.

### Determination of biofilm formation

Solid surface-associated biofilm formation was estimated by the crystal violet staining method with some modifications [25]. A single colony of *B. cereus* 0–9 and its transformants were inoculated into 5 mL LB medium and incubated overnight at 30 °C. Approximately 30 μL of the overnight culture was inoculated into 3 mL MSgg medium in glass culture tubes with a diameter of 0.7 cm. The tubes were incubated in an upright position at 30 °C for 5 days before surface pellicles and cultures were carefully removed from the tubes. The remaining cells and matrices in each tube were stained with 3.5 mL of 0.1% (w/v) crystal violet solution for 20 min at 25 °C. After washing three times with distilled water, the crystal violet attached to the biofilm was solubilized in 3.5 mL of 10% (w/v) SDS and quantified in 200 μL of the solution by measuring the absorbance at 570 nm. The experiment was repeated five times for each strain.

### Assays of complex colony formation

Colony architecture was analyzed using the method of Diethmaier et al. [10] with slight modifications. Strains of *B. cereus* were precultured in LB to an OD_600_ of 0.6 to 0.8, and 1.0 ml of each culture was pelleted and resuspended in 100 μL sterile supernatant. Approximately 2 μL of each cell suspension was spotted onto an NA plate and incubated at 30 °C for 2 to 3 days. The overall pictures of colonies were photographed using a digital single-lens reflex camera (Canon EOS 6D, Japan), and the close-up of individual colonies was photographed by stereomicroscope (Leica S9I, Germany).

### Real-time quantitative reverse transcription-PCR

Real-time quantitative PCR (RT-qPCR) was used to determine the transcription levels of *sipW*, *calY*, *abrB*, *sinI*, and *spo0A* genes in *B. cereus* 0–9 and Δ*spoVG*. *B. cereus* 0–9 and its derivative strains were cultured in MSgg medium at 30 °C, 220 rpm for 24 h and then harvested. Total RNA was extracted as described previously (Gao et al., 2017) and cDNAs were synthesized using a Fast Quant RT Kit (Tiangen Biochemical Technology, Beijing, China) according to the manufacturer’s instructions. For PCR, a GoTaq qPCR Master Mix kit (Shanghai Promega, Shanghai, China) was utilized and qRT-PCR was performed on a Cycler instrument (Bio-Rad, California, USA) as per *B. cereus* 0–9 the manufacturer’s recommended protocol, using the primers listed in Table S1.

### Statistical evaluations

Vegetative growth curves of the wild-type and mutant strains were generated by plotting the average outcomes (OD_600_) of three experiments for each strain. The differences in biofilm formation were analyzed by one-way analysis of variance (ANOVA) followed by Tukey’s pairwise post-hoc comparisons.

## Results

### SpoVG regulates the sporulation of *B. cereus* 0–9

**Fig. 1:**
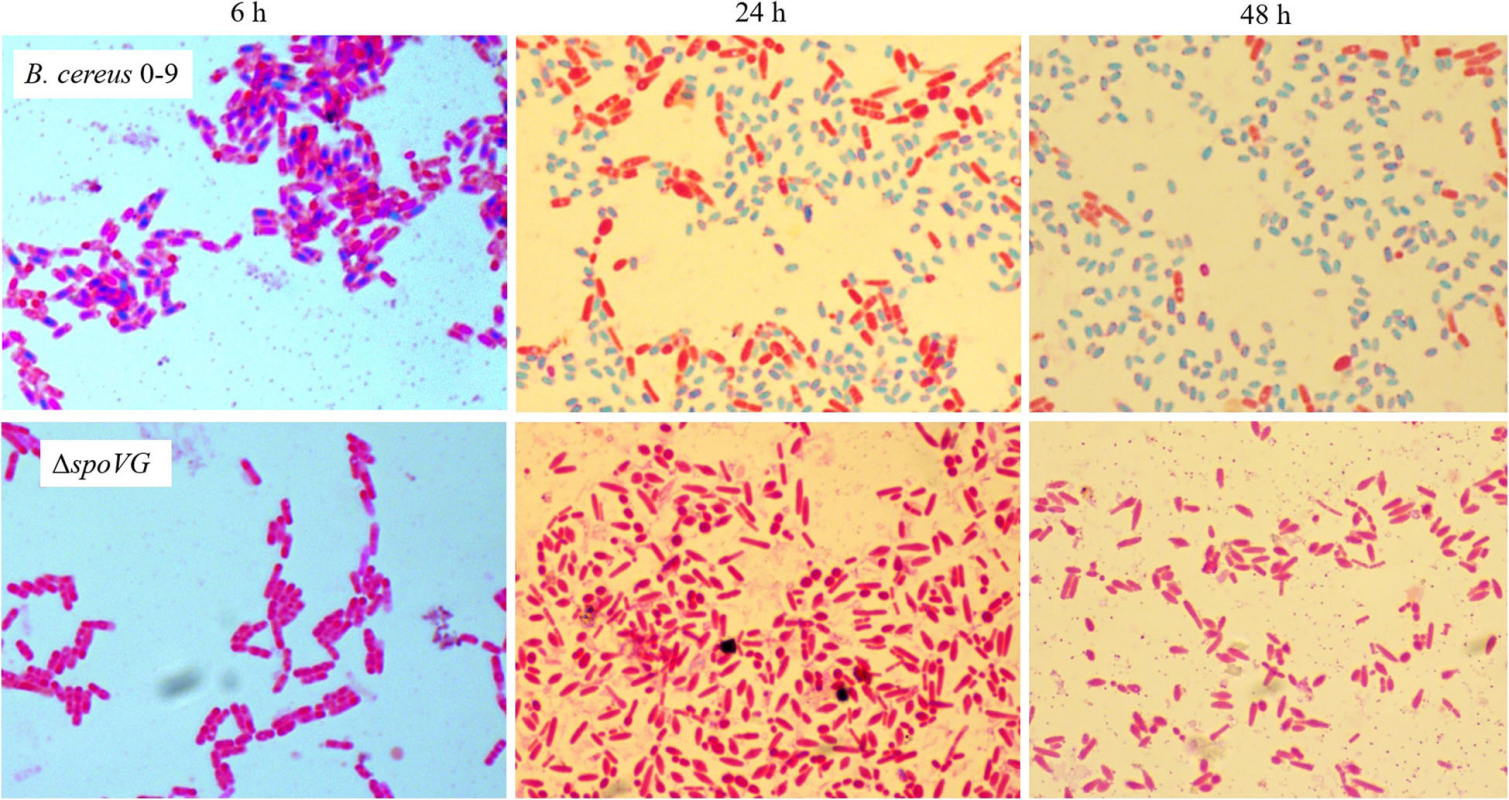
The spore staining results of *B. cereus* 0–9 and its Δ*spoVG* mutant. Tested strains were cultured in MG medium at 30 °C and 220 rpm for 6 h, 24 h and 48 h, respectively. When *B. cereus* 0–9 was stained with malachite green, matured spores will be stained blue, and the cells will be stained red. The cortical defective spores, unable to trap malachite green, will also be stained red by safranin. Thus, for Δ*spoVG* without mature spores, no blue spots were observed after staining under the light microscope

The sporulation process in *B. cereus* 0–9 and its Δ*spoVG* mutant was observed using classic Schaeffer-Fulton staining method [27] following incubation of the strains in MG medium. Deletion of *spoVG* inhibited sporulation of *B. cereus* 0–9 (Fig. 1). After culturing for 6 h, wild-type *B. cereus* 0–9 had formed spore precursors, but the Δ*spoVG* mutant had not. After 24 h, *B. cereus* 0–9 had released a mass of mature spores, but the mutant did not. Further, we determined the spore yield ratio and found Δ*spoVG* mutant could not produce mature spores (Table S2). Our results were quite in accord with recent report of Chen et al. [4] that in *Bacillus anthracis*, whose *spoVG* deletion mutant completely lost sporulation ability and it could not form an asymmetric septum. Therefore, it was concluded that SpoVG protein may play an important role in regulating sporulation of *B. cereus* 0–9.

### SpoVG regulates spore-coat formation in stage V of *B. cereus* 0–9 sporulation

**Fig. 2:**
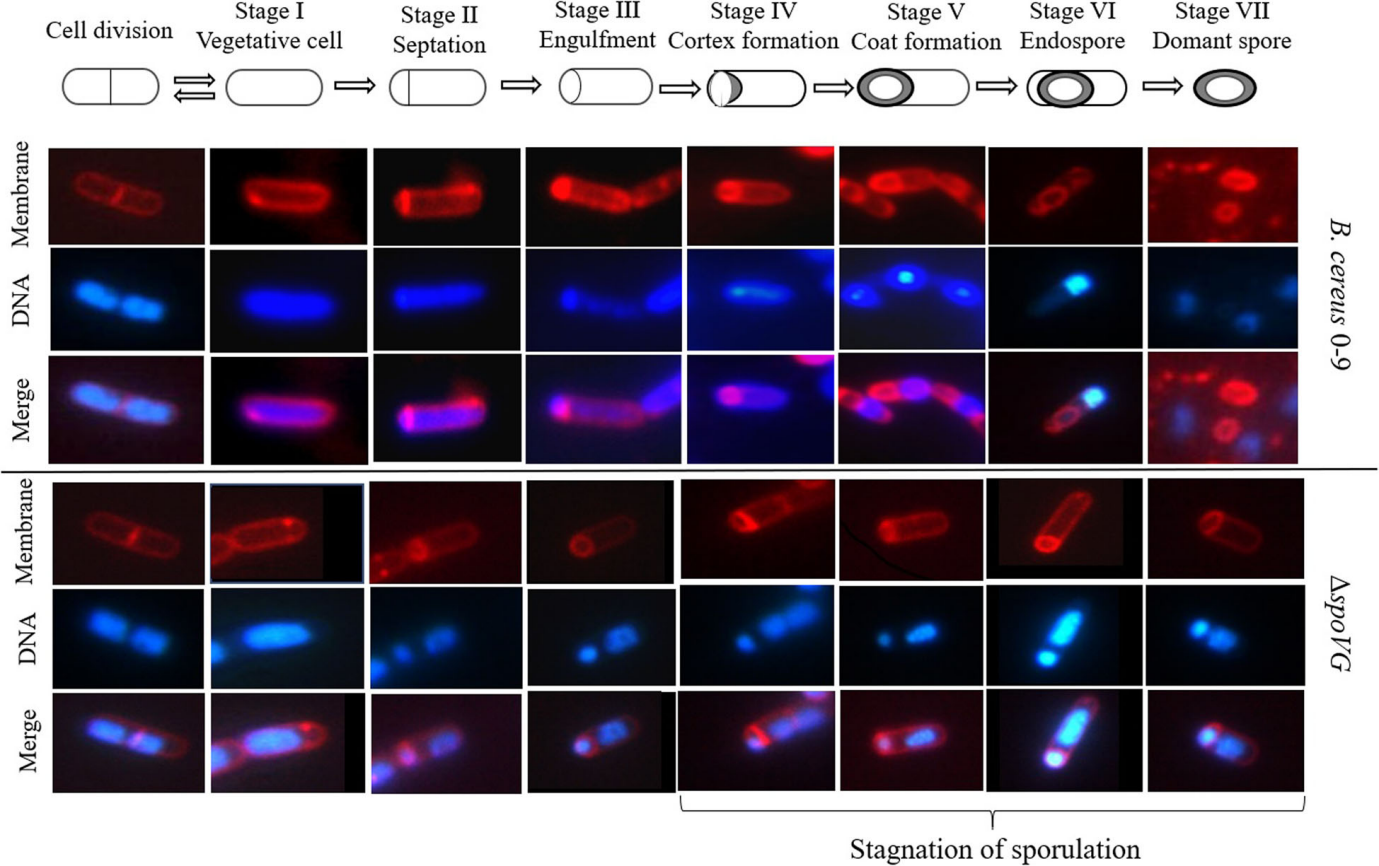
Sporulation of *B. cereus* 0–9 and its Δ*spoVG* mutant. Wild *B. cereus* 0–9 and its Δ*spoVG* mutant were cultured in MG medium at 30 °C and 220 rpm, and stained with two fluorescent dyes. And then, observed under a fluorescence microscope. For the membrane, only the red fluorescence signal of FM4–64 was collected, that is, the cell membrane and spore-coat were stained red; For the cell nucleus, only the blue signal of DAPI is collected, that is, the nuclear DNA is stained blue; And the merge images of the membrane and nuclear DNA showed the overall perspective. The sporulation of Δ*spoVG* mutant doesn’t have the characteristic structure of stage V, it stoped in the stage III-V

To determine the sporulation stage in which SpoVG plays a key role in *B. cereus* 0–9, two distinct fluorescent dyes were used to stain the cell membranes and spores, respectively. The sporulation process in *B. cereus* 0–9 was revealed by fluorescent staining (Fig. 2). There was no significant difference in the process of cell differentiation between *B. cereus* 0–9 and Δ*spoVG* mutant at the first stages (I ~ IV) of culture initiation. After cultured for 8 h, *B. cereus* 0–9 had entered stage V because the red pre-spore coat in the mother cell was visible. After 24 h, we can see clearly that the red spore-precursor turned out in stage VI, and just red dots (mature spores) can be seen in stage VII. However, the sporulation process of the Δ*spoVG* mutant remained at stage IV, with the formation of pre-spores but no outer membranes. We extended the culture time to 48 h, and Δ*spoVG* still stagnated in the stage IV, while mature spores had been released by *B. cereus* 0–9 at this time. This indicated that SpoVG was instrumental in spore-coat formation in stage V of *B. cereus* 0–9 sporulation. These results are consistent with those of *B. subtilis*, but differ from *B. anthracis* where SpoVG protein affects asymmetric septum formation in stage II of sporulation [4]. This may be due to differences in gene expression levels and metabolic regulation mechanisms of bacteria under different environmental and culture conditions.

**Fig. 3:**
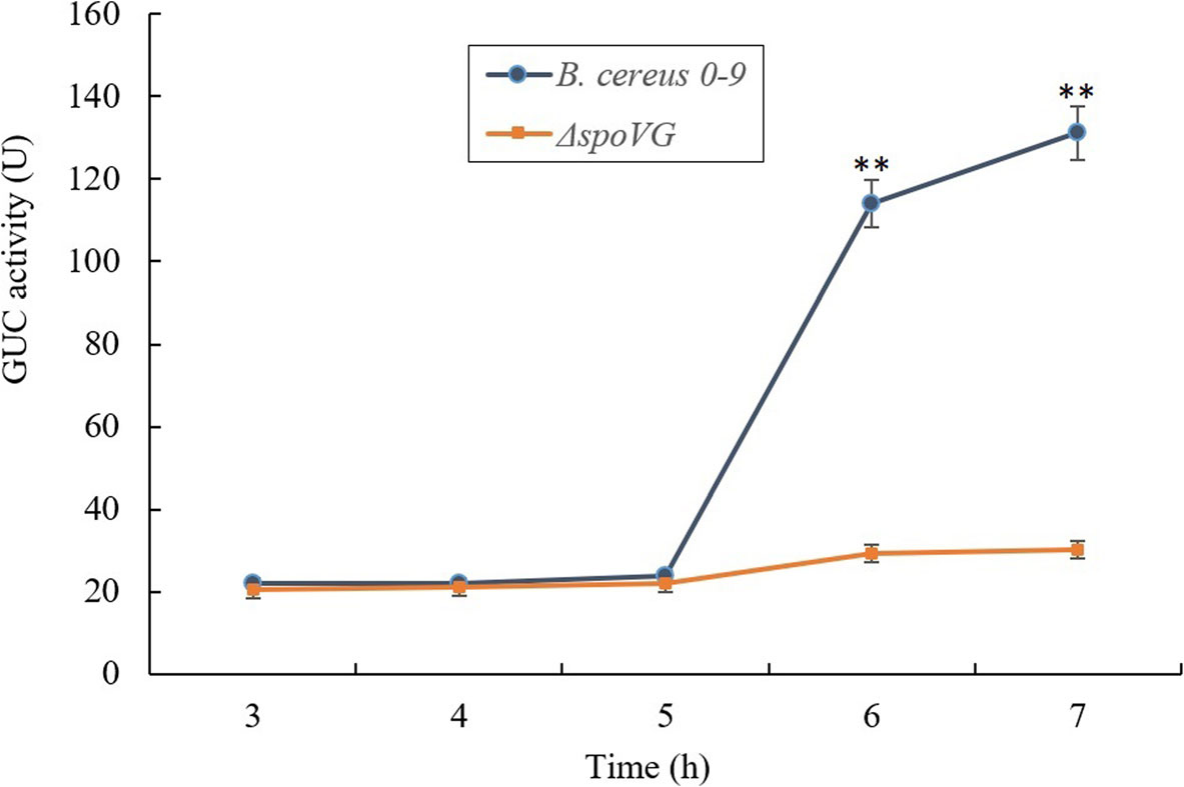
The enzymatic activities of GUS in wild-type *B. cereus* 0–9 and Δ*spoVG* mutant. Test strains were cultured in MG medium for different culturing times. 1 U of GUS was defined as the change of fluorescence intensity per unit of protein in per hour. Each bar represents mean and standard deviations of the mean of all the 3 measurements. “**” means *P* < 0.01 compared with the data of Δ*spoVG* group

The role of SpoVG in stage V of sporulation (the stage of spore-coat formation) was further investigated by constructing two pairs of transcriptional fusion mutants with P*spoIIR*-GUS and P*exsY*-GUS labelling, respectively, in a wild type and *spoVG* background (Table 1). GUS activities of these strains were determined, which reflects the expression level of the gene *spoIIR and exsY*. In the whole cultivation process, the expression levels of *spoIIR* were not different in the wild-type strain and Δ*spoVG* mutant (Fig. S1). Thanks to SpoIIR is a key factor in the regulation of III stage of sporulation, it indicates that *spoVG* gene deletion has no effects on the first three stages of sporulation. But for *exsY*, in the first 5 h, GUS activity was low in both wild type *B. cereus* 0–9 and Δ*spoVG* mutant when they were cultured in MG medium, and there was no significant difference between the wild-type strain and Δ*spoVG* (Fig. 3). However, after culture for 6–7 h, GUS activity of *B. cereus* 0–9 increased by six to seven times, while that of Δ*spoVG* remained unchanged. This indicated that the *spoVG* gene deletion prevented transcription of *exsY*. SpoIIR, required for activation of σ^E^ in the mother cell, is a key factor which plays an important role in the sporulation stage III [28]. ExsY is reported to be a key factor that controls formation of the outer membrane of spores [5]. Thus, SpoVG was concluded to have a critical role in spore-coat formation.

### SpoVG is involved in regulating biofilm formation of *B. cereus* 0–9

**Fig. 4:**
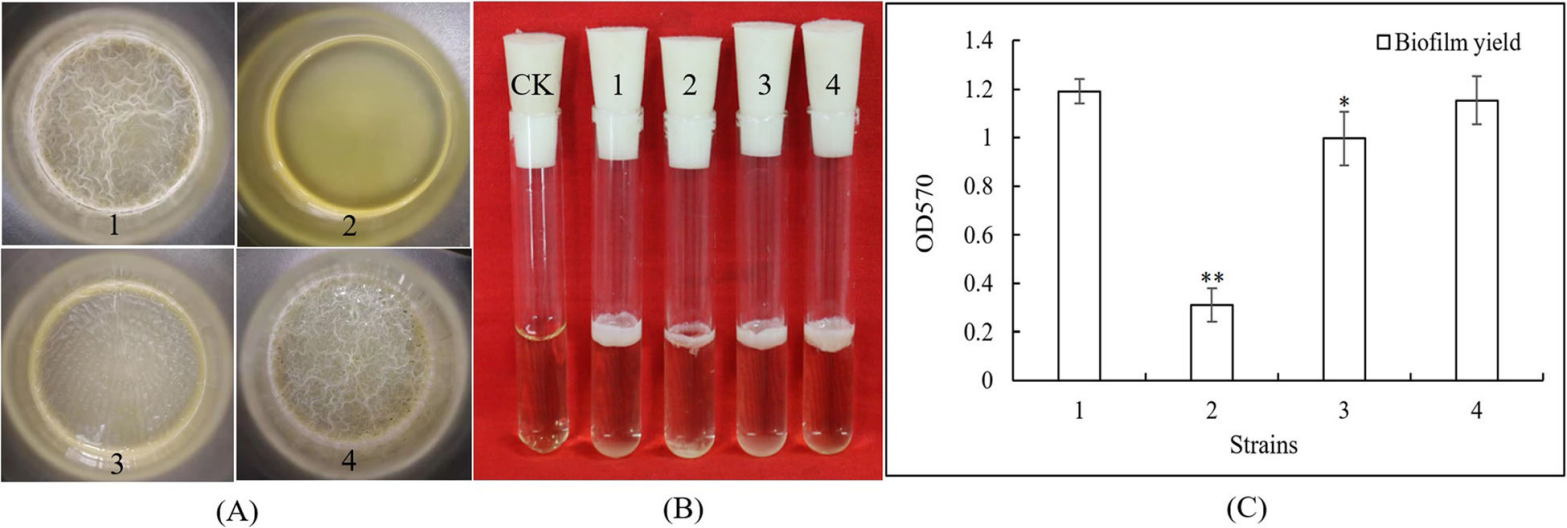
Image and yields of biofilms formed by *B. cereus* 0–9 and its Δ*spoVG* mutants. (A) Top view of the biofilms; (B) Side view of the biofilms in test tube; (C) The quantitative determination results of biofilm yields. Each bar represents the mean of all the measurements and its standard deviations; “*” means 0.01 < *P* < 0.05; “**” means *P* < 0.01. (1) *B. cereus* 0–9; (2) Δ*spoVG*; (3) ∆*spoVG*::*spoVG*_*168*_; (4) ∆*spoVG*::*spoVG* and blank MSgg medium was used for negative control (CK)

The role of SpoVG in biofilm formation of *B. cereus* 0–9 was also investigated. *B. cereus* 0–9 could generate biofilm when it was cultured to the post-stabilization period in MSgg medium, but the Δ*spoVG* mutant did not form wrinkled biofilm on the gas-liquid interface in MSgg medium (Fig. 4, A). To clarify the relationship between *spoVG* and biofilm phenotype, two complemented strains of ∆*spoVG*::*spoVG* and ∆*spoVG*::*spoVG*_*168*_ (Table 1) were constructed using the vector of pMAD*chi*-*spoVG* and pMADchi-*spoVG*_*168*_, respectively, which provided the *spoVG* gene in trans. Complementation of *spoVG* by the native *spoVG* gene (∆*spoVG*::*SpoVG*) fully recovered the biofilm formation abilities of the mutant, while that of the heterologous complement strain ∆*spoVG*::*spoVG*_*168*_ was also restored 83.64%. Quantitative analysis showed that the biofilm yield of the Δ*spoVG* mutant decreased 53.0% compared with that of wild-type *B. cereus* 0–9, whereas biofilm production by the homologous complemented strains ∆*spoVG*::*SpoVG* was not significantly different to wild-type *B. cereus* 0–9. Thus, the gene *spoVG* may be fully reflective of involving in the biofilm formation of *B. cereus* 0–9.

**Fig. 5:**
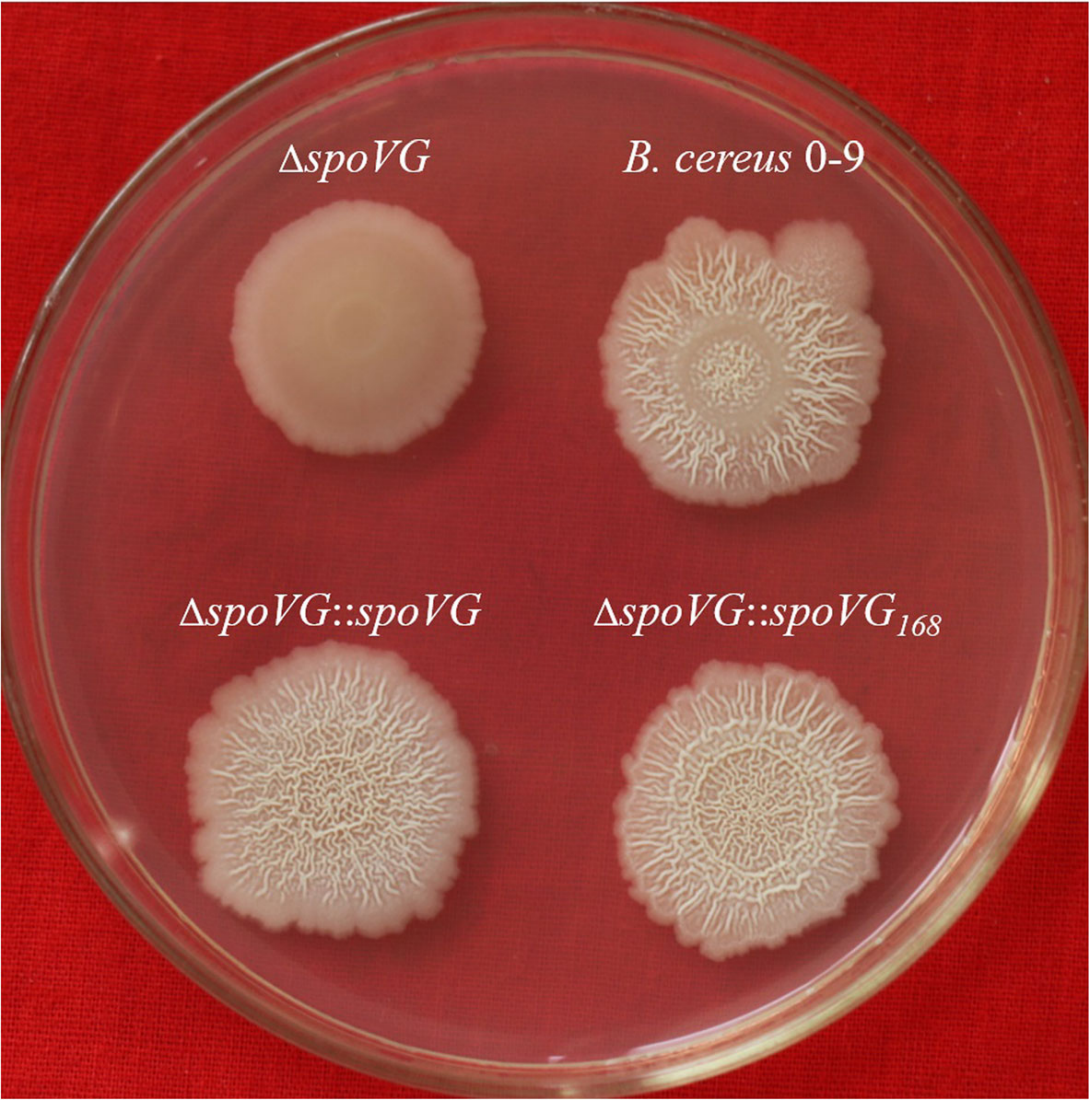
The colonial morphology of *B. cereus* 0–9, Δ*spoVG* and its complemented mutants. Tested strains were seeded on an NA plate and incubated at 30 °C for 2 to 3 days. And then, took photos by digital camera. (1) *B. cereus* 0–9; (2) Δ*spoVG*; (3) ∆*spoVG*::*spoVG*; (4) ∆*spoVG*::*spoVG*_*168*_

Bacteria can form another type of biofilm on solid surfaces, which can be fully reflective of the morphological characteristics of bacterial colonies [2, 29]. Therefore, biofilm formation of the Δ*spoVG* mutant strains was observed on MSgg plate media. Compared with the colony morphology of wild-type *B. cereus* 0–9, colonies of the ∆*spoVG* mutant appeared smooth and the folds on the surface of the wild-type strain completely disappeared in the ∆*spoVG* mutant (Fig. 5). Folds were restored in both ∆*spoVG*::*spoVG* and ∆*spoVG*::*spoVG*_*168*_ strains, demonstrating that both native and exogenous *spoVG* could be used for complementation and restore its phenotype. The folds are a type of biofilm present on solid media and these results were consistent with those obtained by static culture in liquid MSgg medium. This indicated that SpoVG was crucial for biofilm formation of *B. cereus* 0–9.

### SpoVG is involved in regulating both SinI/R and AbrB systems

**Fig. 6:**
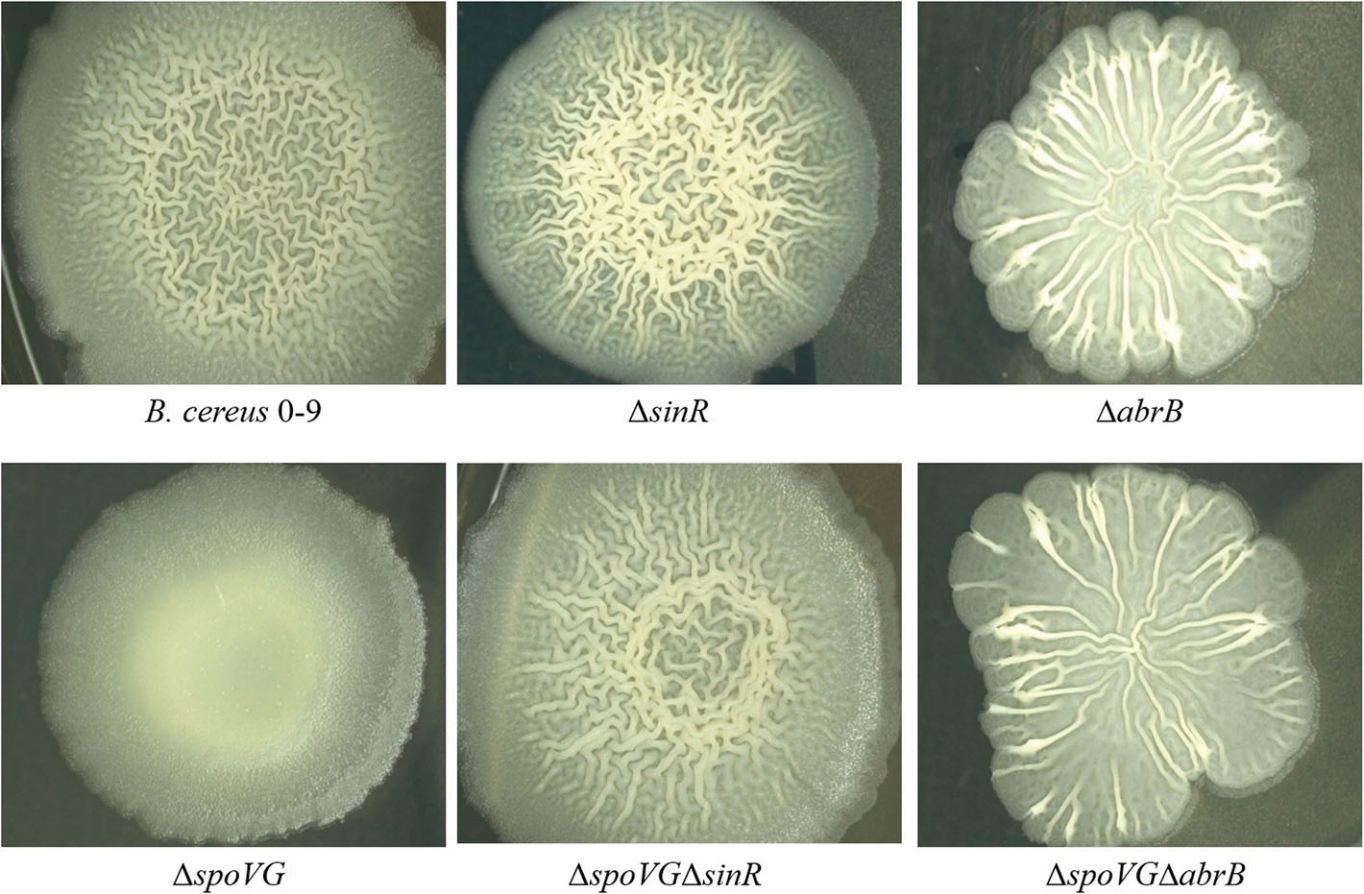
The colonial morphology of *B. cereus* 0–9 and its mutants. Tested strains were seeded on NA plates and incubated at 30 °C for 2 to 3 days. And then, took photos by stereomicroscope. (1) *B. cereus* 0–9; (2) Δ*spoVG*; (3) ∆*abrB*; (4) ∆*spoVG*∆*abrB*; (5) ∆*sinR*; (6) ∆*spoVG*∆*sinR*

To determine whether SpoVG influenced biofilm formation of *B. cereus* 0–9 via SinR or AbrB, two double knockout strains, ∆*spoVG*∆*sinR* and ∆*spoVG*∆*abrB*, were constructed, and their ability to form biofilms on MSgg plate were investigated. The double knockout strain ∆*spoVG*∆*sinR* showed the similar colony morphology as the single knockout strain Δ*sinR* (Fig. 6), that is, the colony surface folds increased meaning the amount of biofilm formation had increased. This indicated that SpoVG is located upstream of *sinR* and likely to play a role in regulating *sinR* gene expression. Furthermore, the double knockout strain ∆*spoVG*∆*abrB* also showed the same colony characteristics as the single mutant Δ*abrB*. For the same reason, it is possible that SpoVG is also located upstream of *abrB* and may also regulate the expression of this gene.

### SpoVG involves in regulating expression of genes related to biofilm formation of *B. cereus* 0–9

The *sipW* and *calY* are two genes closely related to biofilm formation of *B. cereus* 0–9 [9]. To confirm the function of SpoVG protein in the biofilm formation process of *B. cereus* 0–9, the transcription levels of *sipW* and *calY* were measured by qRT-PCR, and the results were shown in Table S3. When cultured statically for 3 days in MSgg medium, transcription levels of *sipW* and *calY* in the Δ*spoVG* mutant were downregulated, compared with levels in wild-type *B. cereus* 0–9. Transcription of *sipW* was decreased 19.97-fold and *calY* was decreased 5.21-fold. These data were consistent with the observed decline of Δ*spoVG* biofilm production and indicated that deletion of *spoVG* gene blocked biofilm formation by suppressing expression of *sipW* and *calY* genes, two positive regulators of biofilm formation. This provides additional confirmation for that SpoVG is an important factor controlling biofilm formation of *B. cereus* 0–9.

**Fig. 7:**
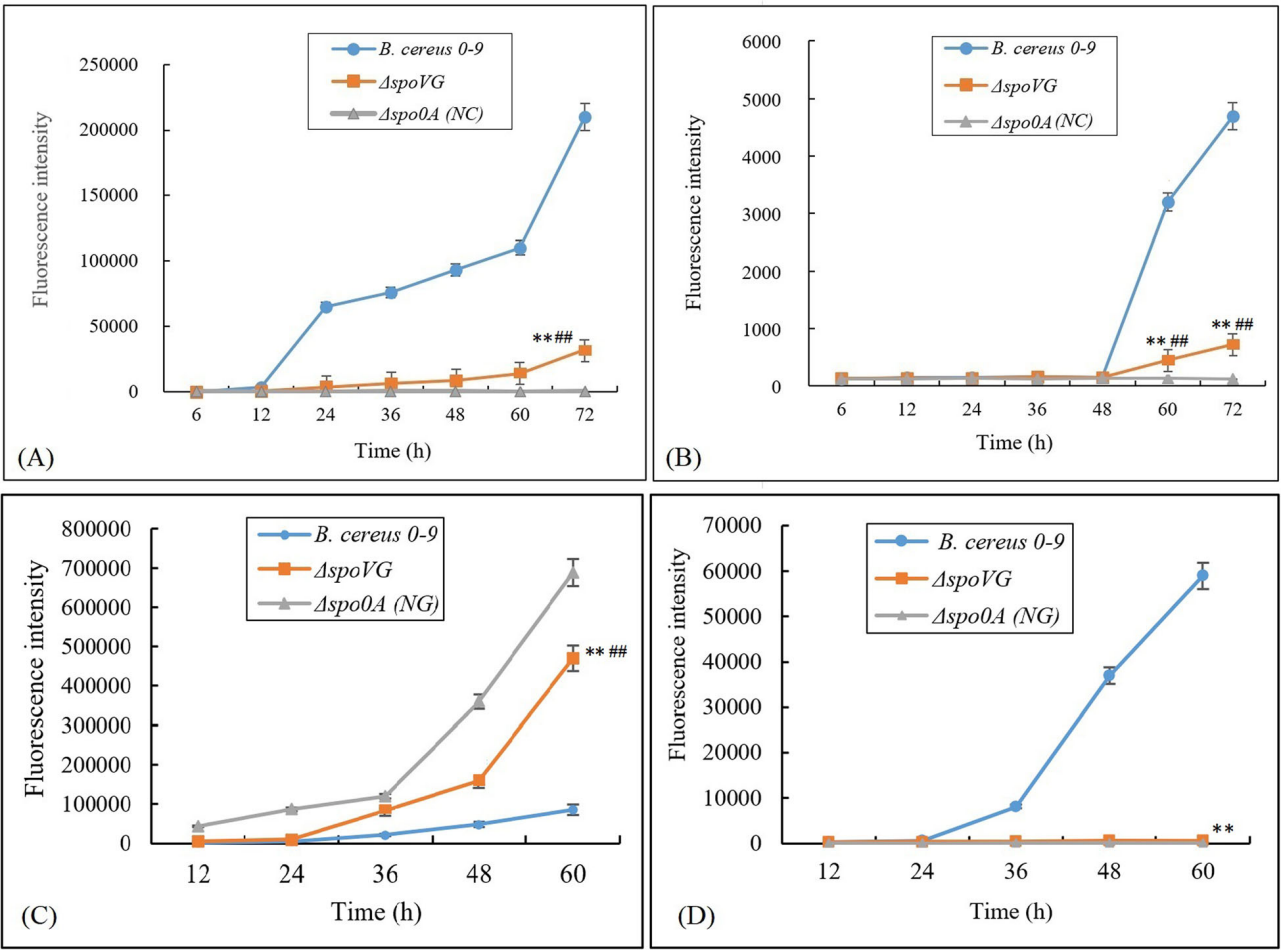
The fluorescence intensity of transcription fusion strains. *B. cereus* 0–9 and Δ*spoVG* harbouring P*sipW*-GFP (**A**), P*calY*-GFP (**B**), P*abrB*-GFP (**C**) and P*sinI*-GFP (**D**), respectively, were constructed in this experiment. The transcription fusion strains of Δ*spo0A* harbouring P*sipW*-GFP, P*calY*-GFP, P*abrB*-GFP and P*sinI*-GFP, respectively, was used for negative control (NC). “**” means *P* < 0.01 compared with the data of *B. cereus* 0–9 group, “**##**” means *P* < 0.01 compared with the NC group

To further determine the role of SpoVG in regulating biofilm formation of *B. cereus* 0–9, several transcriptional fusion strains with P*sipW*-GFP and P*calY*-GFP labelling were constructed; details of the constructed strains are shown in Table 1. These transcriptional fusion strains were cultured in MSgg medium, and at different times, samples were taken. Fluorescence intensity was measured and the results are shown in Fig. 7. The GFP intensity of Δ*spoVG* (with P*sipW*-GFP) declined significantly (*P* < 0.01) comparing with wild-type *B. cereus* 0–9 (with P*sipW*-GFP) after culturing over 12 h (Fig. 7, A). Similarly, the GFP intensity of Δ*spoVG* (with P*calY*-GFP) was significantly (*P* < 0.01) difference from that of wild-type *B. cereus* 0–9 (with P*calY*-GFP) after culturing for 60 h (Fig. 7, B). This suggested that deletion of *spoVG* inhibited transcription of *sipW* and *calY*.

Fluorescence quantitative PCR and transcriptional fusion technology were adopted to study the transcription levels of *abrB* and *sinI* in the Δ*spoVG* mutant. The transcription level of *abrB* increased 11.55-fold, while that of *sinR* increased 17.39-fold in the Δ*spoVG* mutant compared with the wild-type strain of *B. cereus* 0–9. This indicated that SpoVG negatively regulates transcription of *abrB* and *sinR*, but positively regulates transcription of *sinI*. Two additional transcriptional fusion vectors, P*abrB*-GFP and P*sinI*-GFP, were constructed and inserted into the genomes of *B. cereus* 0–9 and the Δ*spoVG* mutant, respectively, to construct four transcriptional fusion strains, designated as wild-type *B. cereus* 0–9 with P*abrB*-GFP, wild-type *B. cereus* 0–9 with P*sinI*-GFP, Δ*spoVG* with P*abrB*-GFP, and Δ*spoVG* with P*sinI*-GFP, and their fluorescence intensity were measured. The expression level of P*abrB*-GFP in Δ*spoVG* mutant was significant (*P* < 0.01) difference from that in wild *B. cereus* 0–9 after culturing for 12 h (Fig. 7, C). However, the expression level of P*sinI*-GFP in Δ*spoVG* mutant decreased significantly (*P* < 0.01) compared with that of wild-type *B. cereus* 0–9 after culturing for 36 h (Fig. 7, D). These results indicate that SpoVG participates in the regulation of biofilm formation through both the AbrB system and the SinI/R system.

### SpoVG affects *B. cereus* 0–9 biofilm formation via positively regulating the transcription of Spo0A

**Fig. 8:**
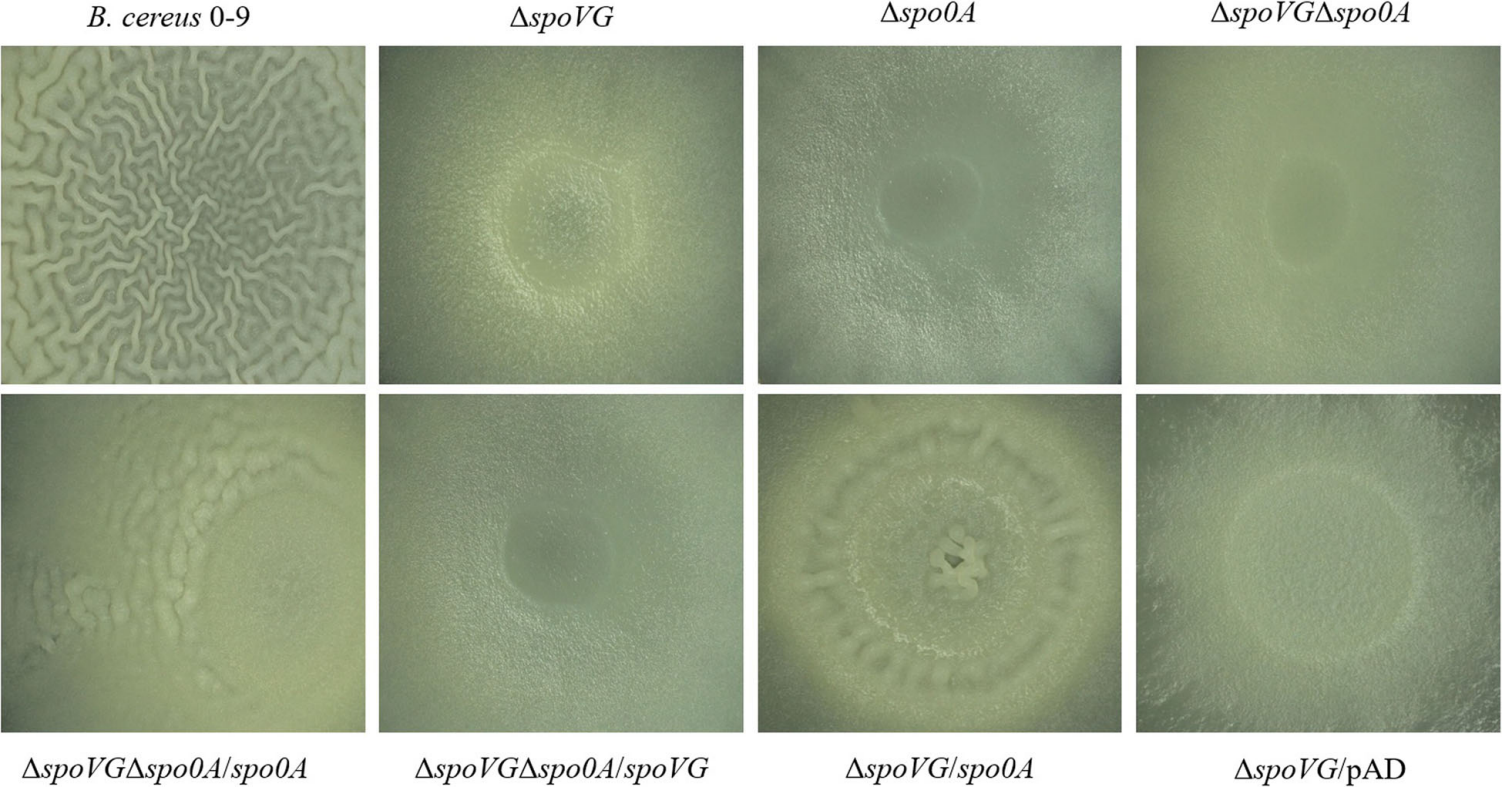
The colonial morphology of *B. cereus* 0–9 and its *spo0A* mutants. Tested strains were seeded on NA medium and cultured at 30 °C for 2 days. And then, the image of each colony was shoot by stereomicroscope. (1) *B. cereus* 0–9; (2) Δ*spo0A*; (3) Δ*spoVG*; (4) Δ*spoVG*Δ*spo0A*; (5) Δ*spoVG*Δ*spo0A*/*spoVG*; (6) Δ*spoVG*Δ*spo0A*/*spo0A*; (7) Δ*spoVG/*Δ*spo0A*; (8) Δ*spoVG/*pAD (Negative Control)

To determine whether SpoVG influenced biofilm formation of *B. cereus* 0–9 via Spo0A, a series of mutant strains were constructed and used to evaluate the regulatory relationship between SpoVG and Spo0A. The colonial morphology of these mutants observed under stereomicroscope were shown in Fig. 8, and their overall appearance under the digital camera is shown in Fig. S2. Colonies of Δs*poVG*, Δs*po0A*, and Δs*poVG*Δs*po0A* were very smooth and had completely lost the folds on their surface (Fig. 8), which was markedly different from the colony morphology of wild-type *B. cereus* 0–9. Therefore, these mutants cannot form normal biofilm on the NA surface. The colony morphology of ∆*spoVG*∆*spo0A*/*spo0A* restored some wrinkle biofilm although its surface is not as densely wrinkled as wild-type *B. cereus* 0–9. However, Δs*poVG*Δs*po0A*/*spoVG* mutant could not restore the biofilm-formation, though the *spoVG* gene was complemented. Thus, we speculated that SpoVG is located upstream of Spo0A in the regulation of biofilm formation and development. But it is very interesting that the colony morphology of ∆*spoVG*∆*spo0A*/*spo0A* was not consistent with that of Δ*spoVG*. We further determined the *spo0A*-overexpressing mutant of Δ*spoVG*/*spo0A*. As a result, if *spo0A* was overexpressed in the Δ*spoVG* strain, the wrinkled biofilm recovered (Fig. 8). This indicated that the absence of SpoVG can depress the transcription of Spo0A, and consequently the regulatory systems of biofilm formation cannot be activated. Together these results demonstrate that SpoVG is located upstream of Spo0A, and SpoVG is likely to participate in regulating biofilm formation of *B. cereus* 0–9 through Spo0A.

**Fig. 9:**
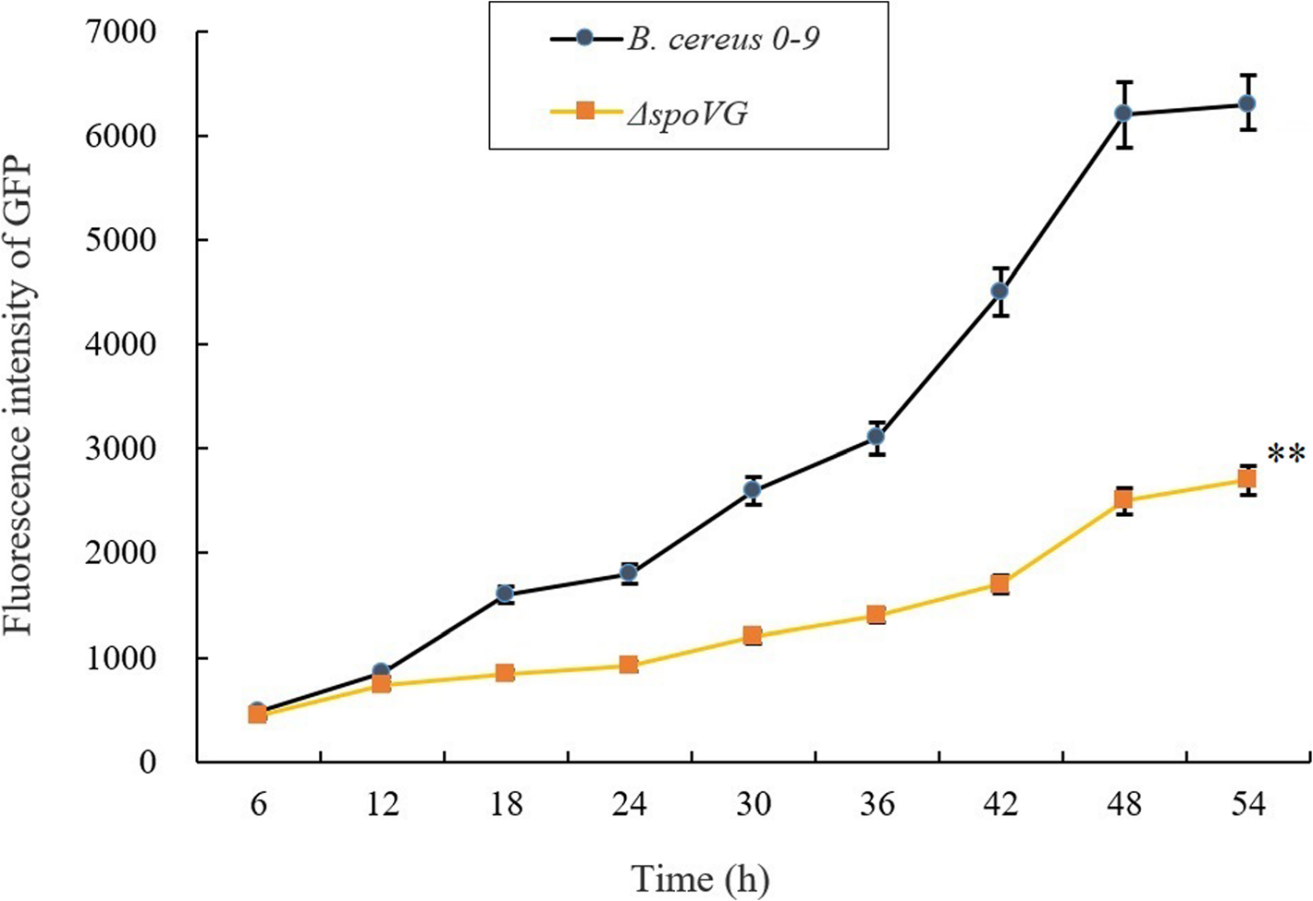
The fluorescence intensity of the transcription fusion strains. Wild *B. cereus* 0–9 with P*spo0A*-GFP and Δ*spoVG* with P*spo0A*-GFP were stationarily cultured in MSgg medium at 30 °C. The fluorescence intensity of the tested strains was measured every 6 h. “**” means *P* < 0.01 compared with the data of *B. cereus* 0–9 group

To further study whether SpoVG can regulate Spo0A, we determined the transcription level of *spo0A* in the Δ*spoVG* mutants by qRT-PCR, and showed the results in Table S3. Compared to wild-type *B. cereus* 0–9, the transcription level of *spo0A* decreased 12.82-fold in the Δ*spoVG* mutants. Deletion of *spoVG* gene inhibited the transcription of *spo0A*. This indicates that SpoVG has a regulatory effect on Spo0A. The fluorescence assay of transcriptional fusion strains of wild-type *B. cereus* 0–9 (with P*spo0A*-GFP) and Δ*spoVG* (with P*spo0A*-GFP) was carried out, and the results (Fig. 9) also showed that *spoVG* gene deletion can inhibited the activity of the promotor of *spo0A*. As previously mentioned, Spo0A can regulate biofilm formation through AbrB and SinI/R system. For another, we determined the sporulation stage in the MSgg medium which is beneficial to stimulate biofilm formation, and found that Δ*spoVG* did not initiate the asymmetric stage of sporulation during the first 36 h of culture, but the wild-type *B. cereus* 0–9 did (Fig. S3 and S4). This result is consistent with the inhibition of the transcription level of Spo0A in the Δ*spoVG* mutant. Therefore, we concluded that SpoVG can regulate *B. cereus* 0–9 biofilm formation through Spo0A.

## Discussion

SpoVG, originally identified in *B. subtilis* as a factor involved in sporulation, is a discrete type of DNA-binding protein commonly found in bacteria [23]. It is broadly conserved, especially among Gram-positive bacteria such as *B. subtilis*, *B. anthracis*, *B. cereus*, *Listeria monocytogenes*, *S. aureus*, and *Clostridium* [4, 29]. SpoVG could alter asymmetric cell division, decreased hemolysin production, and regulated sporulation phenotypes in *B. subtilis* [3]. The homolog of SpoVG in the non-sporulating species *L. monocytogenes* and *S. aureus* was shown to be a modulator of virulence factor synthesis and antibiotic resistance, which may help the organisms coordinate environmental growth and survival [30]. These reports all indicate that SpoVG is a crucial factor for the environmental adaptation of bacteria. *B. cereus* 0–9, a Gram-positive, endospore-forming bacterium isolated from healthy wheat roots by our laboratory, has biological control capacity against several soil-borne plant diseases of wheat. A SpoVG protein (Protein ID: QEF14933.1) encoded by the *spoVG* gene is present in the genome of *B. cereus* 0–9 (GenBank: CP042874.1). The current study aimed to determine whether SpoVG plays an important role in the environmental adaptation of *B. cereus* 0–9.

Despite the discovery of numerous phenotypes of *spoVG* mutants, the function of the SpoVG protein in bacteria has remained unclear. Studies on SpoVG have predominantly focused on *B. subtilis*, *Bacillus thuringiensis*, and *S. aureus*. In *B. subtilis*, SpoVG is mainly involved in stage V of sporulation, that is the formation of the outer membrane of the spore (spore-coat); it negatively regulates asymmetric septum formation and positively regulates cortex formation [3, 4]. SpoVG and SpoIIB jointly determine the sporulation of *B. subtilis*, but single mutants of *spoIIB* or *spoVG* in *B. subtilis* showed only minor effects on sporulation [3, 31]. Confocal microscopy of *B. anthracis* demonstrated that a Δ*spoVG* mutant could not form an asymmetric septum, which is the first morphological change observed during sporulation [4]. SpoVG is highly conserved between *B. anthracis* and *B. subtilis*, but the effects of the Δ*spoVG* mutation on sporulation differ between these two species. Furthermore, the homolog of SpoVG in the non-sporulating species *S. aureus* affected transcription of a number of virulence factors [21, 22]. In the current study, *B. cereus* 0–9 could not form mature spores in the absence of SpoVG (as demonstrated in Fig. 1). The asymmetry diaphragm (Septation, phase II) and endocytosis (Engulfment, phase III) of the sporulation process were not affected, but the stage of cortex formation (Cortex/coat, phase IV and V) was affected (Fig. 2). Initial expression of the gene *exsY*, encoding a major basal layer structural protein (ExsY), occurs when sporulation enters stage V. In the transcriptional fusion assays, the activity of the *exsY* promoter in the transcriptional fusion strain of Δ*spoVG* with PexsY-GUS labeling was decreased (in contrast to wild-type strain), meaning the ability of the P*exsY* promoter was decreased after deletion of the *spoVG* gene. But the activity of *spoIIR* promoter in the transcriptional fusion strain of Δ*spoVG* (with P*spoIIR*-GUS) labeling showed no significant (*P* > 0.05) difference with wild-type strain. Therefore, we concluded that SpoVG plays a key role in the sporulation stage V of *B. cereus* 0–9.

The SpoVG of *B. cereus* 0–9 shared 90.72% homology with that of *B. subtilis* (SpoVG_168_), and 100% sequence identity to SpoVG protein of *B. anthracis* (SpoVG_a_). However, the function of SpoVG in regulating sporulation of *B. cereus* 0–9 is quite accordance to that in *B. subtilis*, but different from that in *B. anthracis*. This difference is probably due to the different metabolic regulation mechanisms of sporulation between different strains. Although both *B. cereus* and *B. anthracis* belong to the *Bacillus cereus* group and have similar genomic structures, they carry different plasmids, and their properties differ due to plasmid content or gene expression associated with key regulatory genes [32]. In *B. anthracis*, there are two virulence plasmid pXO1 (182 kb) and pXO2 (95 kb), which carries the structural gene of toxin protein and the biosynthetic gene of capsular formation, respectively. In *B. cereus* 0–9, there were two plasmids with the size of 514 kb (Accession CP042875) and 14.6 kb (Accession CP042876), and the former has three Rap-Phr quorum sensing systems. Which may be expressed under specific conditions to regulate the phosphorylation of Spo0F in the phosphoric acid transfer system, thus affecting the phosphorylation degree of the main regulatory factor, Spo0A, and affecting spore formation. This regulatory mechanism is similar to that of *B. subtilis*, but not *B. anthracis*. Although the SpoVG protein of *B. cereus* 0–9 was 100% homologous to SpoVG_a_, the regulation mechanism of sporulation is different. Therefore, the sporulation of Δ*spoVG* mutant of *B. cereus* 0–9 stayed on the stage V, and that of *B. anthracis* was blocked before asymmetric septum formation.

Sporulation is usually initiated in harsh conditions, such as nutritional deficiency, high temperatures, and the presence of sanitizer, that make it impossible for bacteria to survive. In the absence of harsh conditions, spore-forming bacteria often combat adverse environmental factors through swarming, synthesis of various extracellular degrading enzymes, and other stress-resistance mechanisms, such as biofilms. Since SpoVG is important in the environmental fitness of bacteria, it is likely to have other functions in addition to regulating sporulation in *B. cereus* 0–9.

*B. cereus* 0–9 has high diversity in lifestyles and ecological niches. It can produce various biofilms that differ in their architecture and mechanism of formation, possibly reflecting an adaptation to varied environments [2]. Biofilm production was investigated and the biofilm formation ability of *B. cereus* 0–9 Δ*spoVG* was markedly decreased compared with that of wild-type *B. cereus* 0–9; this phenotype was restored when the *spoVG* gene was provided in trans. Therefore, it was concluded that SpoVG is closely related to biofilm formation of *B. cereus* 0–9. However, the mechanism as to how *spoVG* regulated biofilm formation was unclear until now. Burke and Portnoya [33] reported that SpoVG was a conserved RNA-binding protein that regulates motility and carbohydrate metabolism, and may positively regulate biofilm formation genes in *L. monocytogenes*, but no supporting evidence was provided. In the *B. cereus* group, regulation of biofilm formation by SpoVG has not previously been reported. It was therefore surprising in the current study that deletion of *spoVG* not only caused spore production inhibition of *B. cereus* 0–9 in MG medium, but also affected biofilm formation of the strain in stationary culture of MSgg medium. Sporulation and biofilm formation of *B. cereus* have been intensively studied, but there is a lack of information on their possible correlation. Therefore, elucidation of the mechanism of SpoVG in regulating biofilm formation, and exploration of possible synergy between biofilm and sporulation may enhance understanding of environmental adaptation of bacteria.

Biofilm formation requires a complex regulatory pathway that coordinates gene expression [7, 8]. The SinI/R system is the most described system for regulating the formation of biofilm in *B. cereus* [11]. In *B. subtilis*, the proteins TapA, TasA, and SipW are required for biofilm formation [34, 35]. Transcription of the *tapA-sipW-tasA* operon is repressed by SinR, a DNA-binding protein [10, 13]. In *B. cereus*, CalY has been described as a cell-surface, membrane-bound zinc metallopeptidase that is active against casein, plasminogen, actin, collagen, or fibrinogen, which are closely related to biofilm formation [34–37]. Based on our previous reports, the SinI/R system and CalY protein exist in *B. cereus* 0–9 and could regulate biofilm formation [25]. The transcription level of *sipW* and *calY* genes were determined by RT-qPCR in the current study. Both *sipW* and *calY* were downregulated when the *spoVG* gene was deleted. Furthermore, construction and analysis of the GFP transcriptional fusion strains (Table 1) demonstrated that activity levels of the promoters of *sipW* and *calY* decreased when *spoVG* was deleted. These results indicated that SpoVG could regulate biofilm formation of *B. cereus* 0–9 through the SinI/R system. Moreover, colony morphology of ∆*spoVG*∆*sinR* were identical to that of the Δ*sinR* mutant, which confirmed the findings of the transcriptional-fusion assays, and indicated that *spoVG* is located upstream of *sinR*. Together, these data suggest that SpoVG regulates biofilm formation of *B. cereus* 0–9 through the SinR system.

According to previous reports, *B. subtilis* forms biofilms in a process that is negatively controlled by the transcription factor AbrB [38]. To determine whether the same process occurred in *B. cereus*, GFP transcription fusion strains were constructed and their green fluorescence intensity was measured during the culturing process. Activity of the *abrB* promoter in Δ*spoVG* mutant was higher than that in wild strain after culturing for 12 h. Further RT-qPCR results revealed that *abrB* gene expression in the ∆*spoVG* strain was upregulated 11.55-fold compared with that of the wild-type *B. cereus* 0–9. Moreover, the colony fold phenotype of the Δ*abrB* strain formed on the plate surface was distinct from that of wild-type *B. cereus* 0–9, while the colony surface folds of the double mutant Δ*spoV*GΔ*abrB* were identical to those of the single deletion mutant Δ*abrB* (Fig. 6). This indicated that *spoVG* is located upstream of *abrB*, and that SpoVG participates in the regulation of biofilm formation through negative regulation of AbrB in *B. cereus* 0–9. Meanwhile, the transcription level of *sinR* was upregulated 17.39-fold in Δ*spoVG* mutant compared with that of wild-type *B. cereus* 0–9. SinR is a key biofilm master repressor, and SinI is a small protein antagonist of SinR [39]. The genes *sinI* and *sinR* exist in tandem in the genome of *B. cereus* and share the same promoter located upstream of *sinI* [16]. The upregulated transcription level of *sinR* could de-repress the biofilm formation [10]. These data from the literature, combined with those from the current study, further confirm that SpoVG is involved in regulating biofilm formation of *B. cereus* 0–9 through the AbrB and SinI/R systems.

Spo0A, a member of the response regulator family of transcriptional regulators, can be activated by multiple histidine kinases, sensing environmental and physiological signals, to regulate the biofilm formation of *B. subtilis* and *B. cereus* [16, 38, 40] (Fig. 8). Spo0A also activates biofilm formation by increasing production of SinI [40]. The role of Spo0A in biofilm formation is to negatively regulate AbrB, which is another transcription factor that negatively regulates biofilm formation of *B. subtilis* [38]. To further explore the systemic mechanism of the involvement of SpoVG in regulation of biofilm formation, the direct regulatory relationship between SpoVG and Spo0A was investigated. The biofilm fold of ∆*spoVG*∆*spo0A* strain was eliminated, but it could be recovered by complementation of *spo0A* gene alone, such as that of ∆*spoVG*∆*spo0A*/*Spo0A* mutant. However, the biofilm was not restored by complementation of *spoVG* gene, such as ∆*spoVG*∆*spo0A*/*SpoVG* (Fig. 8). This proved that *spoVG* was located upstream of *spo0A.* Based on the process of biofilm development in *B. cereus* 0–9, *spoVG* is located upstream of *spo0A* and regulates the expression of Spo0A. Furthermore, it is surprising to observe that the biofilm development of ∆*spoVG*∆*spo0A*/*spo0A* was not identified with Δ*spoVG*. If *spo0A* is overexpressed in ∆*spoVG*, the biofilm recovers, therefore, colony morphology of ∆*spoVG*/*spo0A* is consistent with that of ∆*spoVG*∆*spo0A*/*spo0A* mutant. It may due to the overexpression of the reverse complementary vector of pAD-spo0A. These results indicate that SpoVG may be a regulatory factor of Spo0A in the process of *B. cereus* 0–9 biofilm formation. SpoVG has been reported to play a role in the spore-coat formation stage of sporulation, while Spo0A was reported to be involved in the initial stage of spore differentiation, that is, *spoVG* is located downstream of *spo0A* and is regulated by Spo0A [4, 18, 38]. But our conclusion is contrary to these reports. This may be due to different metabolic regulation mechanisms in *B. cereus* 0–9 under different culture conditions. In germination medium, ∆*spoVG* did not form mature spores and can initiated sporulation but stop them in the stage VI. However, the biofilm production of ∆*spoVG* was inhibited in the static cultivation of MSgg medium. Therefore, we further explored the regulatory relationship between SpoVG and Spo0A in the biofilm-forming condition.

According to the previous report of Zhu et al. [41], SpoVG is a negative regulator and directly represses the expression of *sasC*, which could specifically bind to the promoter region of *sasC* in *S. aureus*. Thus, we examined whether SpoVG affects the transcription of Spo0A. RT-qPCR analysis further demonstrated that the transcription level of *spo0A* in the ∆*spoVG* strain decreased 12.82-folds compared with that in wild-type *B. cereus* 0–9 in MSgg medium. For another, we constructed the transcriptional fusion strains of *B. cereus* 0–9 and Δ*spoVG* with P*spo0A*-GFP, respectively, in the biofilm-forming condition. The determination of fluorescence intensity shows that *spoVG* gene deletion inhibited the activity of promoter of Spo0A. These results indicate that the absence of SpoVG reduces the expression level of Spo0A, and consequently the regulating systems of biofilm formation cannot be activated. Therefore, we conclude that SpoVG participates in biofilm formation of *B. cereus* 0–9 may through regulating the transcription level of *spo0A*.

Spo0A is a well-known transcriptional factor required for early sporulation [42]. Since biofilm formation is also regulated by Spo0A, this indicates that sporulation and biofilm development may be intrinsically linked in *Bacillus*. Intertwined regulatory pathways between biofilm formation and sporulation have been proposed for *B. subtilis* [8], and could be similar in *B. cereus* [40]. It is reported that the bacterial cells have the option of taking either the sporulation or biofilm development pathways upon activation of Spo0A [38]. When Spo0A ~ P levels are relatively low during the early stages of starvation, biofilm is formed, but sporulation does while the Spo0A ~ P levels are high [43]. Thus, we determined the sporulation stage in the biofilm-forming condition, and found that Δ*spoVG* did not initiate the asymmetric stage of sporulation during the first 36 h of culture, but the wild-type *B. cereus* 0–9 did (Fig. S3 and S4). This indicated that *spoVG* gene mutation also inhibited the spore differentiation of *B. cereus* 0–9 under this culture condition. In *B. thuringiensis*, three distinct cell types controlled by quorum-sensing regulators have been identified: PlcR-controlled virulent cells, NprR-dependent necrotrophic cells, and cells committed to sporulation [44]. In the developmental stages of *B. thuringiensis*, virulence, necrotrophism, and sporulation processes are three distinct physiological pathways controlled by Rap phosphatases and Spo0A-P [44]. In another related development, gradual accumulation of Spo0A ∼ P is essential for the proper temporal order of the Spo0A regulon expression, and that reduction in sporulation efficiency results from the reversal of that order (Monika et al., 2013). It is thus possible that the phosphorylation level of Spo0A ∼ P is a key regulatory mechanism that controls the initiation of biofilm formation or sporulation by *B. cereus* 0–9. This mechanism is so interesting that it will attract us to explore it in our further work.

## Conclusions

As a biocontrol bacterium derived from soil, *B. cereus* 0–9 has evolved various environmental adaptation mechanisms, such as swarming motility, biofilm formation, spore differentiation, etc. SpoVG is a regulator that is broadly conserved, especially among many Gram-positive bacteria, and may help the organisms coordinate environmental growth and to survival. SpoVG regulates stage V of sporulation in *B. cereus* 0–9, which is identical to the process in *B. subtilis*, but differs to that of *B. anthracis*. Since SpoVG plays a crucial role in environmental fitness of bacteria, it is likely to have other functions in addition to regulating sporulation in *B. cereus* 0–9. Biofilm production of Δ*spoVG* mutants were therefore studied and SpoVG was demonstrated to be involved in regulating biofilm formation of *B. cereus* 0–9. Transcriptional fusion strains, double gene mutant strains and their complemented mutants were used to explore the mechanism of SpoVG regulation of biofilm formation in *B. cereus* 0–9, and the results consistently showed that SpoVG regulated biofilm formation of *B. cereus* 0–9 through both the AbrB and SinI/R systems. Analysis of the direct regulatory relationship between SpoVG and Spo0A led to the conclusion that SpoVG is located upstream of Spo0A and participates in regulation of biofilm formation of *B. cereus* 0–9 through regulating the transcription level of *spo0A*. This study demonstrates that SpoVG is an important regulator of Spo0A, which is crucial for both sporulation and biofilm formation of *B. cereus* 0–9. SpoVG is therefore important for the environmental adaptability of *B. cereus*. The findings of this study enhance understanding of bacterial biofilms and may facilitate the development new biological control strategies utilizing bacteria.

## Supplementary Information

**Additional file 1: Table S1.** Primers used in this text. **Table S2**. The spore yield rate of experiment strains with different culture time. **Table S3.** Gene expression level of *sipW*, *calY*, *sinR* and *abrB* in *B. cereus* 0–9 and Δ*spoVG* determined by qRT-PCR. **Figure S1**. The GUS enzymatic activities of wild *B. cereus* 0–9 and Δ*spoVG* mutant at MG medium under different culturing times. One U of GUS was defined as the change of fluorescence intensity per unit of protein in per hour. Each bar represents mean and standard deviations of the mean of all the 3 measurements. **Figure S2.** The colonial morphology of *B. cereus* 0–9 and its *spo0A* mutants. Tested strains were seeded on NA medium and cultured at 30°C for 2 days. And then, the image of each colony was shoot by digital camera. (1) *B. cereus* 0–9; (2) Δ*spo0A*; (3) Δ*spoVG*; (4) Δ*spoVG*Δ*spo0A*; (5) Δ*spoVG*Δ*spo0A*/*spoVG*; (6) Δ*spoVG*Δ*spo0A*/*spo0A*; (7) Δ*spoVG/*Δ*spo0A*; (8) Δ*spoVG/*pAD (Negative Control). **Figure S3.** Sporulation of *B. cereus* 0–9 when it was cultured in MSgg medium at 30 °C, 220 rpm for 24 h, 36 h, 42 h, 48 h and 56 h, respectively, and stained with two fluorescent dyes. And then, observed under a fluorescence microscope. For the membrane, only the red fluorescence signal of FM4–64 was collected, that is, the cell membrane and spore-coat were stained red; For the cell nucleus, only the blue signal of DAPI is collected, that is, the nuclear DNA is stained blue; And the merge images of the membrane and nuclear DNA showed the overall perspective. **Figure S4**. Sporulation of Δ*spoVG* mutant when it was cultured in MSgg medium at 30 °C, 220 rpm for 24 h, 36 h, 42 h and 48 h, respectively, and stained with two fluorescent dyes. And then, observed under a fluorescence microscope. For the membrane, only the red fluorescence signal of FM4–64 was collected, that is, the cell membrane and spore-coat were stained red; For the cell nucleus, only the blue signal of DAPI is collected, that is, the nuclear DNA is stained blue; And the merge images of the membrane and nuclear DNA showed the overall perspective.

## Data Availability

Data on the genomes of *B. cereus* 0–9 have been submitted to the Gene Bank of NCBI which is open, and its GenBank ID is CP042874.1. The website is as follow: *https://www.ncbi.nlm.nih.gov/nuccore/CP042874.1/* And all the other data generated during this study are included in this published article [and its supplementary information files.

## Abbreviations

MG: Modified germination
NA: Nutrient agar
MSgg: Minimal salts glycerol glautamate
MU: 4-methylumbelliferyl
MUG: 4-methylumbelliferyl beta-D-glucuronide dihydrate
GFP: Green fluorescent protein
qRT-PCR: Real-time quantitative PCR
CFU: Colony-forming units

## References

[1] Moons P, Chris M, Abram A (2009). Bacterial interactions in biofilms. Crit Rev Microbiol.

[2] Majed R, Faille C, Kallassy M, Gohar M (2016). *Bacillus cereus* biofilms-same, only different. Front Microbiol.

[3] Matsuno K, Sonenshein AL (1999). Role of SpoVG in asymmetric septation in *Bacillus subtilis*. J Bacteriol.

[4] Chen M, Lyu Y, Feng E, Zhu L, Pan C, Wang D (2020). SpoVG is necessary for sporulation in *Bacillus anthracis*. Microorganisms..

[5] Boone TJ, Mallozzi M, Nelson A, Thompson B, Khemmani M, Lehmann D (2018). Coordinated assembly of the *Bacillus anthracis* coat and exosporium during bacterial spore outer layer formation. Mol Biol Physiol.

[6] Hagan AK, Plotnick YM, Dingle RE, Mendel ZI, Cendrowski SR, Sherman DH (2018). Petrobactin protects against oxidative stress and enhances sporulation efficiency in *Bacillus anthracis* Sterne. Mol Biol Physiol.

[7] Romero D, Vlamakis H, Losick R, Kolter R (2014). Functional analysis of the accessory protein TapA in *Bacillus subtilis* amyloid fiber assembly. J Bacteriol.

[8] Vishnoi M, Narula J, Devi SN, Dao HA, Igoshin OA, Fujita M (2013). Triggering sporulation in *Bacillus subtilis* with artificial two-component systems reveals the importance of proper Spo0A activation dynamics. Mol Micro.

[9] Zhang J, Wang H, Xie T, Huang Q, Xiong X, Liu Q (2020). The YmdB protein regulates biofilm formation dependent on the repressor SinR in *Bacillus cereus* 0-9. World J Microbiol Biotechnol.

[10] Diethmaier C, Newman JA, Akos TK, Kaever V, Stülke J (2014). The YmdB phosphodiesterase is a global regulator of late adaptive responses in *Bacillus subtilis*. J Bacteriol.

[11] Newman JA, Rodrigues C, Lewis RJ (2013). Molecular basis of the activity of SinR protein, the master regulator of biofilm formation in *Bacillus subtilis*. J Biol Chem.

[12] Branda SS, Gonzalez-Pastor JE, Ben-Yehuda S, Losick R, Kolter R (2001). Fruiting body formation by *Bacillus subtilis*. Proc Natl Acad Sci U S A.

[13] Kearns DB, Chu F, Branda SS, Kolter R, Losick R (2005). A master regulator for biofilm formation by *Bacillus subtilis*. Mol Microbiol.

[14] Lewis RJ, Brannigan JA, Smith I, Wilkinson AJ (1996). Crystallisation of the *Bacillus subtilis* sporulation inhibitor SinR, complexed with its antagonist. SinI FEBS Lett.

[15] Lewis RJ, Brannigan JA, Offen WA, Smith I, Wilkinson AJ (1998). An evolutionary link between sporulation and prophage induction in the structure of a repressor: anti-repressor complex. J Mol Biol.

[16] Xu S, Yang N, Zheng S, Yan F, Jiang C, Yu Y, et al. The spo0A-sinI-sinR regulatory circuit plays an essential role in biofilm formation, nematicidal activities, and plant protection in *Bacillus cereus* AR156. MPMI. 2017. 10.1094/MPMI-02-17-0042-R. 10.1094/MPMI-02-17-0042-R 28430084

[17] Hamon MA, Stanley NR, Britton RA, Grossman AD, Lazazzera BA (2004). Identification of AbrB-regulated genes involved in biofilm formation by *Bacillus subtilis*. Mol Microbiol.

[18] Rosenbluh A, Banner CDB, Losick R, Fitz-James PC (1981). Identification of a new developmental locus in *Bacillus subtilis* by construction of a deletion mutation in a cloned gene under sporulation control. J Bacteriol.

[19] Liu X, Zhang S, Sun B (2016). SpoVG regulates cell wall metabolism and oxacillin resistance in methicillin-resistant *Staphylococcus aureus* strain N315. Antimicrob Agents Chemother.

[20] Savage CR, Jutras BL, Bestor A, Tilly K, Rosa PA, Tourand Y (2018). *Borrelia burgdorferi* SpoVG DNA- and RNA-binding protein modulates the physiology of the Lyme disease spirochete. J Bacteriol.

[21] Meier S, Goerke C, Woiz C, Seidl K, Homerova D, Schulthess B (2007). Sigma(B) and the sigma(B)-dependent *arlRS* and *yabJ*-*spoVG* loci affect capsule formation in *Staphylococcus aureus*. Infect Immun.

[22] Schulthess B, Bloes DA, Francois P, Girard M, Schrenzel J, Bischoff M (2011). The sigma(B)-dependent *yabJ-spoVG* operon is involved in the regulation of extracellular nuclease, lipase, and protease expression in *Staphylococcus aureus*. J Bacteriol.

[23] Jutras BL, Chenail AM, Rowland CL, Dustin C, Clarke MM, Tomasz B (2013). Eubacterial SpoVG homologs constitute a new family of site-specific DNA-binding proteins. PLoS One.

[24] Zhang J, Wang H, Huang Q, Zhang Y, Zhao L, Liu F (2020). Four superoxide dismutases of *Bacillus cereus* 0-9 are non-redundant and perform different functions in diverse living conditions. World J Microbiol Biotechnol.

[25] Zhang J, Li M, Zhang Y, Sang L, Liu Q, Zhao L, et al. GapB is involved in biofilm formation dependent on LrgAB but not the SinI/R system in *Bacillus cereus* 0-9. Front Microbiol. 2020. 10.3389/fmicb.2020.591926. 10.3389/fmicb.2020.591926 PMC7750190 33365021

[26] Xu Y, Chen M, Zhang Y, Wang M, Ying W, Huang Q (2014). The phosphotransferase system gene *ptsI* in the endophytic bacterium *Bacillus cereus* is required for biofilm formation, colonization, and biocontrol against wheat sharp eyespot. FEMS Microbio Let.

[27] Oktari A, Supriatin Y, Kamal M, Syafrullah H (2017). The bacterial endospore stain on Schaeffer-Fulton using variation of methylene blue solution. J Phys Conf Ser.

[28] Hilbert DW, Piggot PJ. Compartmentalization of gene expression during *Bacillus subtilis* spore formation. Microbiol mol biol. 2004;68:234–62. 10.1128/MMBR.68.2.234-262.2004 PMC419919 15187183

[29] Liu J, Prindle A, Humphries J, Gabalda-Sagarra M, Asally M, Lee DD (2015). Metabolic co-dependence gives rise to collective oscillations within biofilms. Cell..

[30] Bischoff M, Brelle S, Minatelli S, Molle V (2016). Stk1-mediated phosphorylation stimulates the DNA-binding properties of the *Staphylococcus aureus* SpoVG transcriptional factor. Biochem Biophys Res Commun.

[31] Londono-Vallejo JA, Frehel C, Stragier P (1997). SpoII Q, a forespore-expressed gene required for engulfment in *Bacillus subtilis*. Mol Microbiol.

[32] Ehling-Schulz M, Koehler TM, Lereclus D (2019). The *Bacillus cereus* group: *Bacillus species* with pathogenic potential. Microbiol Spectr.

[33] Burke TP, Portnoya DA (2017). SpoVG is a conserved RNA-binding protein that regulates *Listeria monocytogenes* lysozyme resistance, virulence, and swarming motility. mBio.

[34] Candela T, Fagerlund A, Buisson C, Gilois N, Kolstø A, Økstad O (2019). CalY is a major virulence factor and a biofilm matrix protein. Mol Microbiol.

[35] Caro-Astorga J, Pérez-García A, De Vicente A, Romero D (2015). A genomic region involved in the formation of adhesin fibers in *Bacillus cereus* biofilms. Front Microbiol.

[36] Fricke B, Drossler K, Willhardt I, Schierhorn A, Menge S, Rucknagel P (2001). The cell envelope-bound metalloprotease (camelysin) from *Bacillus cereus* is a possible pathogenic factor. Biochim Biophys Acta.

[37] Grass G, Schierhorn A, Sorkau E, Muller H, Rucknagel P, Nies DH (2004). Camelysin is a novel surface metalloproteinase from *Bacillus cereus*. Infect Immun.

[38] Hamon MA, Lazazzera BA (2001). The sporulation transcription factor Spo0A is required for biofilm development in *Bacillus subtilis*. Mol Microbiol.

[39] Bai U, Mandic-Mulec I, Smith I (1993). SinI modulates the activity of SinR, a developmental switch protein of *Bacillus subtilis*, by protein-protein interaction. Genes Dev.

[40] Huang Y, Flint SH, Palmer JS (2020). *Bacillus cereus* spores and toxins – the potential role of biofilms. Food Microbiol.

[41] Zhu Q, Liu B, Sun B (2020). SpoVG modulates cell aggregation by regulating *sasC* expression and eDNA. Appl Environ Microbiol.

[42] Burbulys D, Trach KA, Hoch JA (1991). Initiation of sporulation in *B subtilis* is controlled by a multicomponent phosphorelay. Cell.

[43] Fujita M, Losick R. Evidence that entry into sporulation in *Bacillus subtilis* is governed by a gradual increase in the level and activity of the master regulator Spo0A. Genes DevGene Dev. 2005;19:2236–44. 10.1101/gad.1335705 PMC1221893 16166384

[44] Verplaetse E, Slamti L, Gohar M, Lereclus D (2017). Two distinct pathways lead *Bacillus thuringiensis* to commit to sporulation in biofilm. Res Microbiol.

