# Supplementary material for: SpoVG is an important regulator of sporulation and affects biofilm formation by regulating Spo0A transcription in Bacillus cereus 0–9
Source: BMC Microbiol. 2021 Jun 8;21:172. doi: 10.1186/s12866-021-02239-6 (PMC8186074; doi:10.1186/s12866-021-02239-6)
Supplement: Supplementary file 1 — Additional file 1: Table S1. Primers used in this text. Table S2. The spore yield rate of experiment strains with different culture time. Table S3. Gene expression level of sipW, calY, sinR and abrB in B. cereus 0–9 and ΔspoVG determined by qRT-PCR. Figure S1. The GUS enzymatic activities of wild B. cereus 0–9 and ΔspoVG mutant at MG medium under different culturing times. One U of GUS was defined as the change of fluorescence intensity per unit of protein in per hour. Each bar represents mean and standard deviations of the mean of all the 3 measurements. Figure S2. The colonial morphology of B. cereus 0–9 and its spo0A mutants. Tested strains were seeded on NA medium and cultured at 30°C for 2 days. And then, the image of each colony was shoot by digital camera. (1) B. cereus 0–9; (2) Δspo0A; (3) ΔspoVG; (4) ΔspoVGΔspo0A; (5) ΔspoVGΔspo0A/spoVG; (6) ΔspoVGΔspo0A/spo0A; (7) ΔspoVG/Δspo0A; (8) ΔspoVG/pAD (Negative Control). Figure S3. Sporulation of B. cereus 0–9 when it was cultured in MSgg medium at 30 °C, 220 rpm for 24 h, 36 h, 42 h, 48 h and 56 h, respectively, and stained with two fluorescent dyes. And then, observed under a fluorescence microscope. For the membrane, only the red fluorescence signal of FM4–64 was collected, that is, the cell membrane and spore-coat were stained red; For the cell nucleus, only the blue signal of DAPI is collected, that is, the nuclear DNA is stained blue; And the merge images of the membrane and nuclear DNA showed the overall perspective. Figure S4. Sporulation of ΔspoVG mutant when it was cultured in MSgg medium at 30 °C, 220 rpm for 24 h, 36 h, 42 h and 48 h, respectively, and stained with two fluorescent dyes. And then, observed under a fluorescence microscope. For the membrane, only the red fluorescence signal of FM4–64 was collected, that is, the cell membrane and spore-coat were stained red; For the cell nucleus, only the blue signal of DAPI is collected, that is, the nuclear DNA is stained blue; And the merge images of th [file 12866_2021_2239_MOESM1_ESM.docx]

Table S1 Primers used in this text.

| Name | Sequence (5′-3′) | Application |
| --- | --- | --- |
| *spoVG-*up-BamHI-s | ACACGGATCCTGAAACTTTAGAAAGTGAGGCA | Used for construction of Δ*spoVG* mutant |
| *spoVG-*up-XhoI-a | CACACTCGAGTTCGTAAAATTCGAATGAAAAG |  |
| *spoVG-*d-XhoI-s | CACACTCGAGGTCACTTCCATCTTGTGTTCAC |  |
| *spoVG-*d-EcoRI-a | CACAGAATTCGCTGGCGGAACAATTCAAGGTA |  |
| *spo0A*-up-BamHI-s | TTCCGGATCCGCTTATCAAAAGCACTAGGT | Used for construction of Δ*spo0A* mutant |
| *spo0A*-up-XbaI-a | ACCCTCTAGAAAGCTGAGACTTGAACATAA |  |
| *spo0A*-down-XbaI-s | CCCATCTAGAAGACATACTTTAATTTTCTC |  |
| *spo0A*-down-SalI-a | GTGTGTCGACATTGGAGATATGATTACTG |  |
| *abrB*-up-BamHI-s | ACACGGATCCTTTCAACAAAATGAAAAGGGAG | Used for construction of Δ*abrB* mutant |
| *abrB*-up-KpnI-a | CACAGGTACCTATATCGAAACAGCAAAATAAG |  |
| *abrB*-down-KpnI-s | ACACGGTACCAACAATACCAGTAGATTTCATA |  |
| *abrB*-down-xhoI-a | ACACCTCGAGTGTACAGAATACCAACCTTCAT |  |
| SpovG-ORF-BamHI-s | ACACGGATCCATGGAAGTGACTGACGTAAGATTAC | Used for construction of complementary strains |
| *spovG*-XhoI-a | CACACTCGAGTTACGAAGCACCTGCTTCTTCA |  |
| 0-9-pMAD-chi-s | TTTACTAACAATCGCTTCAGGC | Used for construction of complementary strains |
| 0-9-pMAD-chi-a | TTGCAAGGTTGATATTGTCCGTTA |  |
| Pro-spovG-EcoRI-s | ACACGAATTCAAGGACATGGATGATTTTAATG |  |
| spovG-XhoI-a | CACACTCGAGTTACGAAGCACCTGCTTCTTCA |  |
| *168spoVGcom-MluI-s* | ACACACGCGTACGGACAATATTTTGACACTCA | Used for construction of heterologous complementary strains |
| *168spoVGcom-XhoI-a* | CACACTCGAGTTAAGAAGCTCCAGCTTCTTCG |  |
| *spo0Acom-SalI-s* | ACCCGTCGACTATATATTATTAGCTCGCTT | Used for construction of heterologous complementary strains |
| *spo0Acom-BamHI-a* | AACCGGATCCTCCCCTTCTCCCTATCTATATCT |  |
| *abrB-Qpcr-s* | AGGACGCTCTTGAAATCTATG | Quantification of *abrB* expression |
| *abrB-Qpcr-a* | AAGAAAGGTTACCGTCAGATA |  |
| c*alY*-Qpcr-s | CATCAGCAGCATTGGGGTTA | Quantification of *calY* expression |
| c*alY*-Qpcr-a | CTACAAGCGTCTTAGCCTTT |  |
| *sipW*-Qpcr-s | ATCACTCACCGTATTATCG | Quantification of *sipW* expression level |
| *sipW*-Qpcr-a | CTTTCCAACTACATTTTCAG |  |
| 16sRNA-s | ACTGGGACTGAGACACGG | Reference genes |
| 16sRNA-a | GATAACGCTTGCCACCTA |  |
| P*sipw*-BamHI-s | CACAGGATCCGTGAAAGTAATAAGTGTTGGGA | Used for construction of fluorescently labeled strain for the P*sipw* gene of *B. cereus* 0-9 |
| P*sipw*-*gfp*-a | AGTTCTTCTCCTTTACTCATCGTCTCTCTCCCTCTCCGTTGTTTTA |  |
| *gfp*-P*sipW*-s | AACGGAGAGGGAGAGAGACGATGAGTAAAGGAGAAGAACTTTTCAC |  |
| *gfp*-XhoI-a | CACACTCGAGTTATTTGTATAGTTCATCCATGCCA |  |
| *PcalY*-BamHI-s | ACACGGATCCAGGGAACGTTAGCCCTCACCTC | Used for construction of fluorescently labeled strain for the P*calY* gene of *B. cereus* 0-9 |
| *PcalY*-*gfp*-a | AGTTCTTCTCCTTTACTCATCACAATCAATTCCCCCTAGCT |  |
| *gfp*-*calY*-s | GCTAGGGGGAATTGATTGTGATGAGTAAAGGAGAAGAACTTTTCAC |  |
| gfp-XhoI-a | CACACTCGAGTTATTTGTATAGTTCATCCATGCCA |  |
| *abrB-gfp-*BamHI*-s* | ACACGGATCCTAGGTCTTAATGCTCAATATCT | Used for construction of fluorescently labeled strain for the P*abrB* gene of *B. cereus* 0-9 |
| *abrB-gfp-* | AGTTCTTCTCCTTTACTCATAATTTTTTTCCTCCTAAATAAATAGT |  |
| *gfp-abrB-s* | TATTTAGGAGGAAAAAAATTATGAGTAAAGGAGAAGAACTTTTCAC |  |
| *gfp*-XhoI-a | CACACTCGAGTTATTTGTATAGTTCATCCATGCCA |  |
| P*sinI*-BamHI-s | ACACGGATCCCAATATTTAGTCATATTATGTA | Used for construction of fluorescently labeled strain for the P*sinI* gene of *B. cereus* 0-9 |
| P*sinI*-*gfp*-a | AGTTCTTCTCCTTTACTCATATGTAATTCCTCCCTAATTATCGGTC |  |
| *gfp*-P*sinI*-s | TAATTAGGGAGGAATTACATATGAGTAAAGGAGAAGAACTTTTCAC |  |
| *gfp*-XhoI-a | CACACTCGAGTTATTTGTATAGTTCATCCATGCCA |  |

Table S2

Table S2 The spore yield rate of experiment strains with different culture time.

| Incubation time | 6 h | | |  | 24 h | | |  | 48 h | | |
| --- | --- | --- | --- | --- | --- | --- | --- | --- | --- | --- | --- |
|  | Total count of viable bacteria（cfu/ml） | Spore count（cfu/ml） | Spore ratio |  | Total count of viable bacteria（cfu/ml） | Spore count（cfu/ml） | Spore ratio |  | Total count of viable bacteria（cfu/ml） | Spore count（cfu/ml） | Spore ratio |
| 0-9 | 85ｘ10^5^ | 0 | 0 |  | 197ｘ10^5^ | 185ｘ10^5^ | 0.94 |  | 123ｘ10^5^ | 114ｘ10^5^ | 0.92 |
| ∆*spoVG* | 79ｘ10^5^ | 0 | 0 |  | 190ｘ10^6^ | 0 | 0 |  | 119ｘ10^6^ | 0 | 0 |

Table S3 Gene expression level of *sipW*, *calY*, *sinR* and *abrB* in *B. cereus* 0-9 and Δ*spoVG* determined by qRT-PCR.

| CT value | *B. cereus* 0-9 | Δ*spoVG* | ΔΔct value | 2^-ΔΔct^ value | Gene expression level |
| --- | --- | --- | --- | --- | --- |
| *sipW* | 8.68 | 13.0 | 4.32 | 0.05 | 19.97-fold↓ |
| *calY* | 10.08 | 12.3 | 2.22 | 0. 215 | 5.21-fold↓ |
| *sinR* | 19.96 | 15.84 | -4.12 | 17.39 | 17.39-fold↑ |
| *abrB* | 5.14 | 1.61 | -3.53 | 11.55 | 11.55-fold↑ |
| *Spo0A* | 6.68 | 10.36 | 3.68 | 0.078 | 12.82-fold↓ |

The information of spoVG genes downloaded from GenBank are as follow:

septation protein SpoVG [*Bacillus cereus*]

GenBank: QEF14933.1

GenPept Identical Proteins Graphics

>QEF14933.1 septation protein SpoVG [*Bacillus cereus*]

MEVTDVRLRRVNTEGRMRAIASITLDHEFVVHDIRVIDGNNGLFVAMPSKRTPDGEFRDIAHPINSGTRSKIQDAVLTEYHRLGELEEVEFEEAGAS

>AQR79990.1 Putative septation protein SpoVG [*Bacillus subtilis* *subsp.* *subtilis str.* 168]

MEVTDVRLRRVNTDGRMRAIASITLDHEFVVHDIRVIDGNNGLFVAMPSKRTPDGEFRDITHPINSSTRGKIQDAVLNEYHRLGDTEALEFEEAGAS

>QKU25960.1 septation regulator SpoVG [*Bacillus anthracis*]

MEVTDVRLRRVNTEGRMRAIASITLDHEFVVHDIRVIDGNNGLFVAMPSKRTPDGEFRDIAHPINSGTRSKIQDAVLTEYHRLGELEEVEFEEAGAS

Figure S1


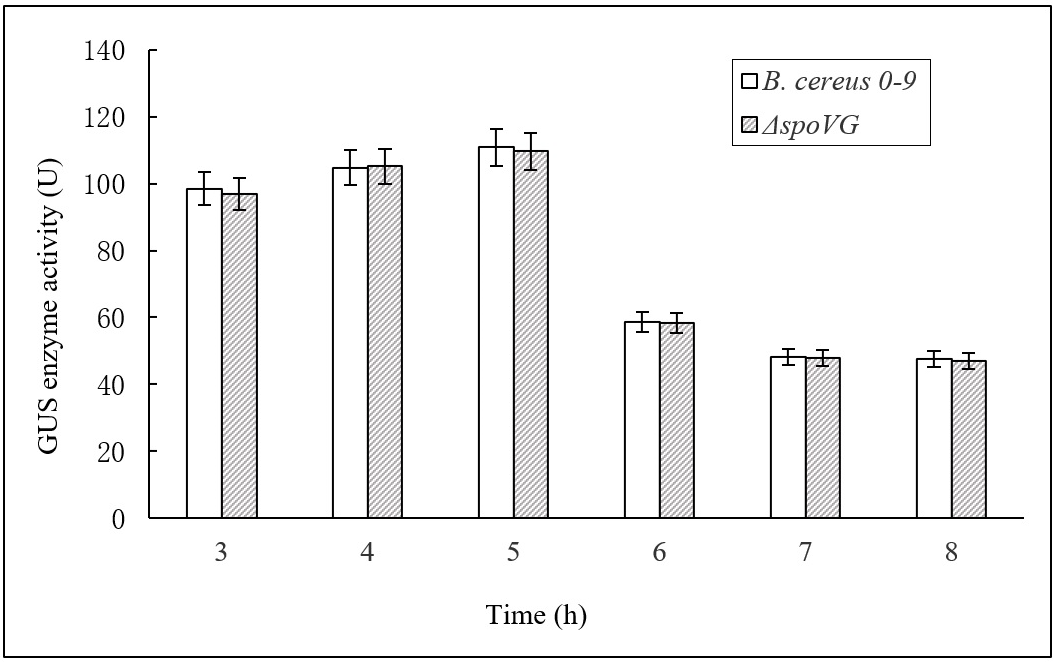


Figure S1 The GUS enzymatic activities of wild *B. cereus* 0-9 and Δ*spoVG* mutant at MG medium under different culturing times. 1U of GUS was defined as the change of fluorescence intensity per unit of protein in per hour. Each bar represents mean and standard deviations of the mean of all the 3 measurements.


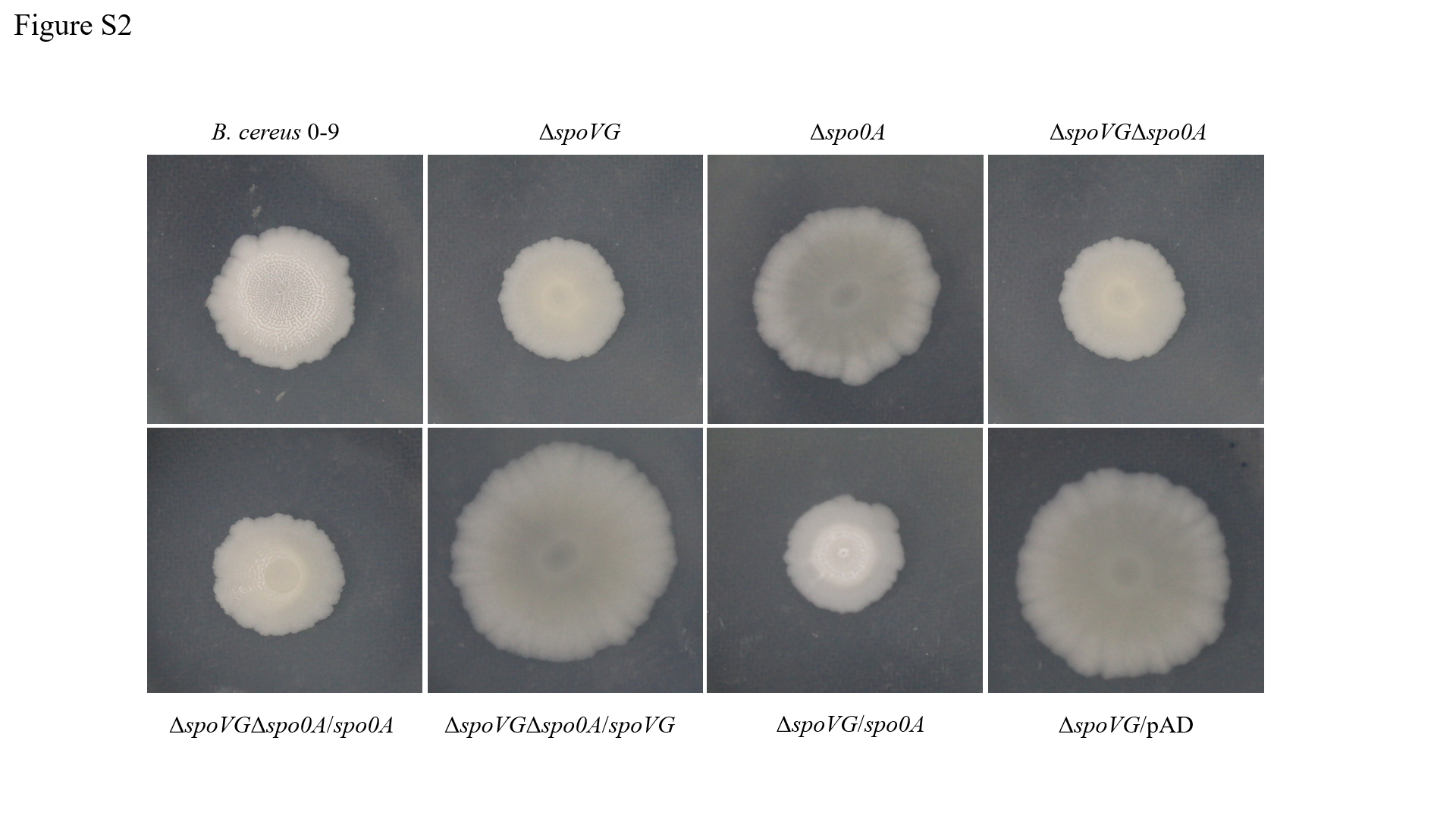


Figure S2 The colonial morphology of *B. cereus* 0-9 and its *spo0A* mutants. Tested strains were seeded on NA medium and cultured at 30℃ for 2 days. And then, the image of each colony was shoot by

digital camera. (1) *B. cereus* 0-9; (2) Δ*spo0A*; (3) Δ*spoVG*; (4) Δ*spoVG*Δ*spo0A*; (5)Δ*spoVG*Δ*spo0A*/*spoVG*; (6) Δ*spoVG*Δ*spo0A*/*spo0A*; (7) Δ*spoVG/*Δ*spo0A*; (8) Δ*spoVG/*pAD (Negative Control).


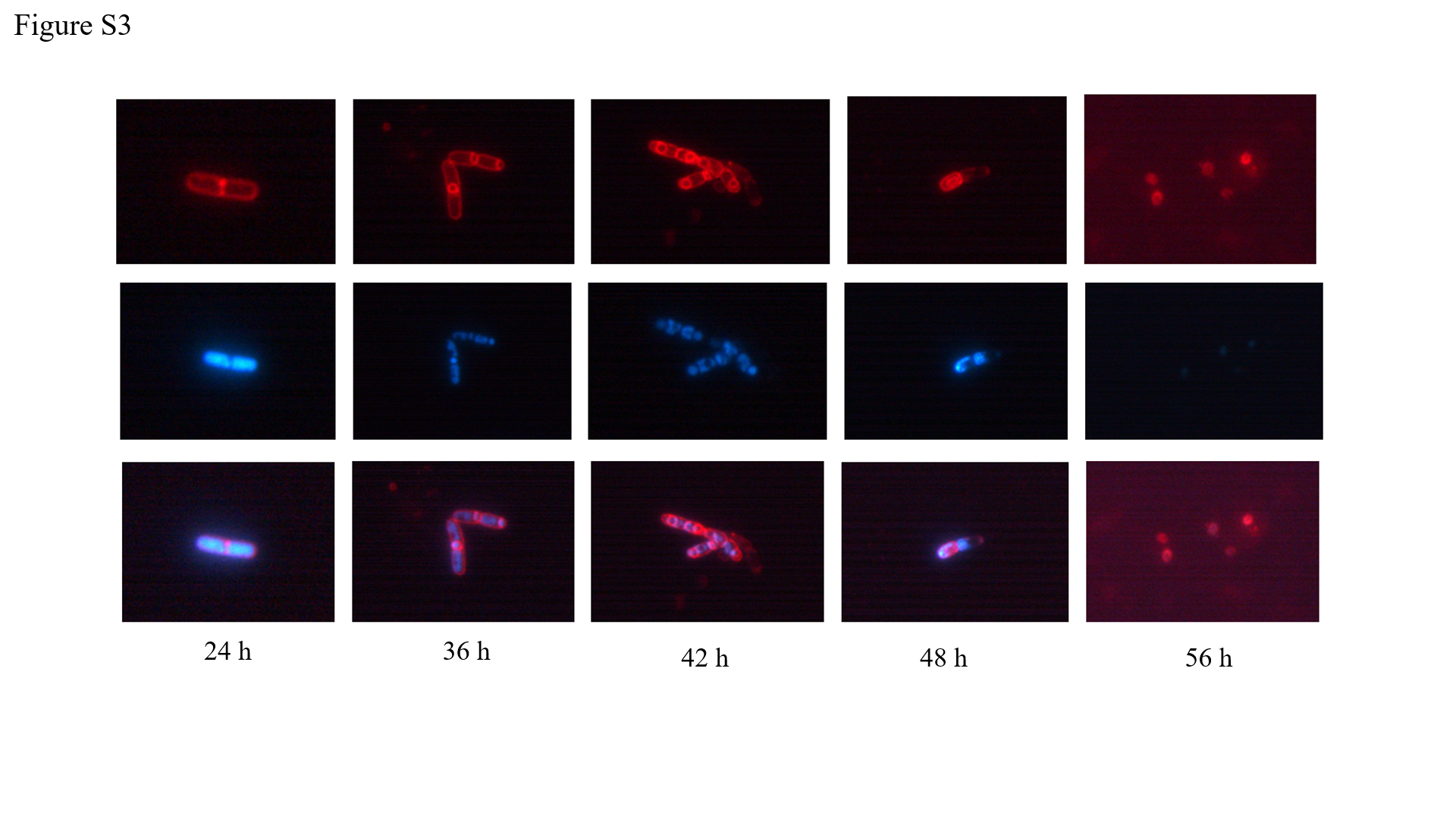


Figure S3 Sporulation of *B. cereus* 0-9 when it was cultured in MSgg medium at 30°C, 220 rpm for 24 h, 36 h, 42 h, 48 h and 56 h, respectively, and stained with two fluorescent dyes. And then, observed under a fluorescence microscope. For the membrane, only the red fluorescence signal of FM4-64 was collected, that is, the cell membrane and spore-coat were stained red; For the cell nucleus, only the blue signal of DAPI is collected, that is, the nuclear DNA is stained blue; And the merge images of the membrane and nuclear DNA showed the overall perspective.


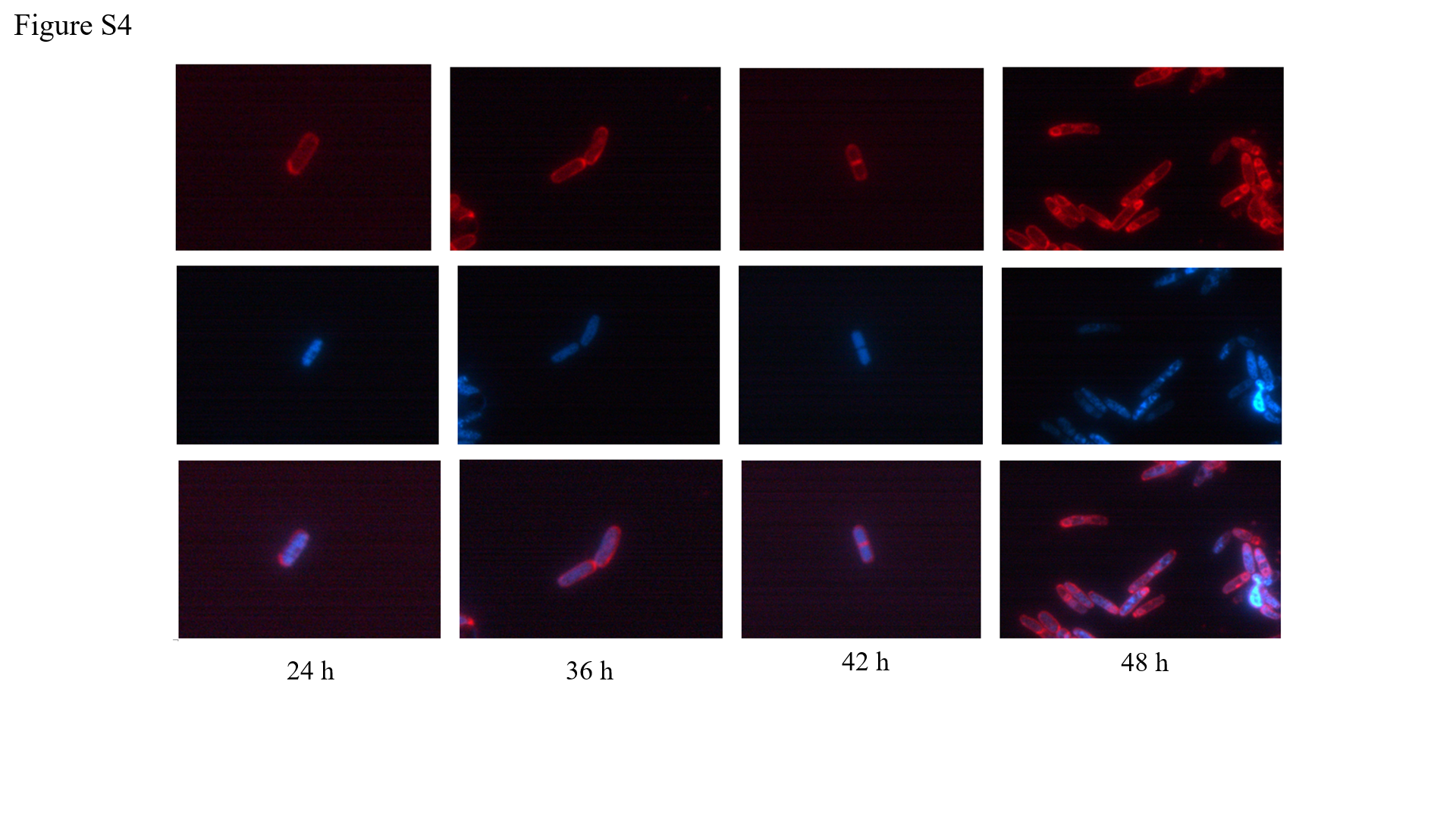


Figure S4 Sporulation of Δ*spoVG* mutant when it was cultured in MSgg medium at 30°C, 220 rpm for 24 h, 36 h, 42 h and 48 h, respectively, and stained with two fluorescent dyes. And then, observed under a fluorescence microscope. For the membrane, only the red fluorescence signal of FM4-64 was collected, that is, the cell membrane and spore-coat were stained red; For the cell nucleus, only the blue signal of DAPI is collected, that is, the nuclear DNA is stained blue; And the merge images of the membrane and nuclear DNA showed the overall perspective.
